# Two New Cinnamyl Isovalerate Derivatives from *Sabina gaussenii*

**DOI:** 10.3390/molecules21050571

**Published:** 2016-04-29

**Authors:** Zhang-Hua Sun, Ning-Hua Tan, Guang-Zhi Zeng, Yu-Mei Zhang

**Affiliations:** 1 Key Laboratory of Tropical Plant Resources and Sustainable Use, Xishuangbanna Tropical Botanical Garden, Chinese Academy of Sciences, Kunming 650223, China; sunzh@gdim.cn; 2 State Key Laboratory of Applied Microbiology Southern China, Guangdong Provincial Key Laboratory of Microbial Culture Collection and Application, Guangdong Open Laboratory of Applied Microbiology, Guangdong Institute of Microbiology, Guangzhou 510070, China; 3 State Key Laboratory of Phytochemistry and Plant Resources in West China, Kunming Institute of Botany, Chinese Academy of Sciences, Kunming 650204, China; nhtan@mail.kib.ac.cn (N.-H.T.); gzh_zeng@mail.kib.ac.cn (G.-Z.Z.)

**Keywords:** *Sabina gaussenii*, cinnamyl isovalerate, cytotoxicity

## Abstract

Chemical investigation of the 90% acetone extract of the branches and leaves of *Sabina gaussenii* led to the isolation of two new cinnamyl isovalerate derivatives (**1**–**2**) and eighteen known compounds (**3**–**20**). Their structures were determined mainly by means of MS, 1D- and 2D-NMR data, and this is the first time these compounds have been reported from this plant. The biological activity test results indicated that the 90% acetone extract showed cytotoxicity against the human lung adenocarcinoma (A549) cell line (IC_50_ = 0.98 ± 0.1 μg/mL), compound **6** showed cytotoxicities against human cervical carcinoma (HeLa) (IC_50_ = 0.4 ± 0.1 μM ) and human gastric carcinoma (BGC-823) (IC_50_ = 0.9 ± 0.2 μM) cancer cell lines, and compound **19** showed cytotoxicities against HeLa (IC_50_ = 1.5 ± 0.4 μM), BGC-823 (IC_50_ = 7.0 ± 0.8 μM ), and A549 (IC_50_ = 10.6 ± 1.5 μM ) cancer cell lines.

## 1. Introduction

*Sabina gaussenii* is endemic to China and is usually used as a hedge plant. The genus *Sabina*, which used to belong to genus *Juniperus*, has about 50 species and spread widely throughout the northern hemisphere [1]. According to the literature, the *Sabina* plants have been reported to be a rich source of bioactive terpenoids [2]. Up to now, only one diterpenoid and a few flavones have been reported from *S. gaussenii* [3]. As part of serial investigations on the Gymnospermae plants and in order to seek more novel bioactive compounds, we carried out an extensive chemical study on *S. gaussenii* [4,5,6,7]. In this paper, we report the isolation and structure elucidation of two new cinnamyl isovalerate derivatives (**1**–**2**) together with eighteen other known compounds (**3**–**20**) from the branches and leaves of *S. gaussenii*, in addition to a screening of their cytotoxicities.

## 2. Results and Discussion

**Figure 1:**
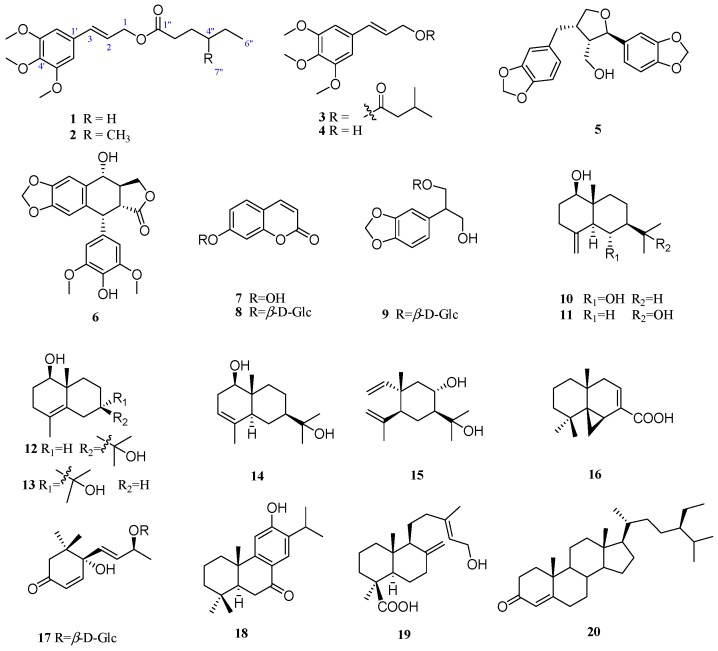
The chemical structures of compounds **1**–**20**.

The air-dried powder of the branches and leaves of *S. gaussenii* was extracted with 90% acetone at room temperature to give a crude extract, which was suspended in H_2_O and successively partitioned with petroleum ether, ethyl acetate (EtOAc), and *n*-butyl alcohol (*n*-BuOH). Column chromatographic separations of these extracts afforded compounds **1**–**20** (Figure 1). The two new structures (**1**–**2**) were identified by spectroscopic analyses and physicochemical properties, while the known compounds were identified as 3’,4’,5’-dimethoxycinnamyl isovalerate (**3**) [8], 3’,4’,5’-dimethoxycinnamyl alcohol (**4**) [9], dihydrosesamin (**5**) [10], 4’-*O*-demethylepipodophyllotoxin (**6**) [11], 7-hydroxy coumarin (**7**) [12], 7-β-d-glucosyloxy coumarin (**8**) [13], 1-β-d-glucosyloxy-2-(3,4-methylenedioxyphenyl)-propane-l,3-diol (**9**) [14], lβ,6α-dihydroxy-4(14)-eudesmene (**10**) [15], selin-4(15)-en-1β, 11-diol (**11**) [16], 4-eudesmene-1β, 11-diol (**12**) [17], 7-*epi*-4-eudesmene-1β, 11-diol (**13**) [17], 3-eudesmene-1β, 11-diol (**14**) [17], 8α,11-elemodiol (**15**) [18], hinokiic acid (**16**) [19], corchoionoside C (**17**) [20], hinokiol (**18**) [21], isocupressic acid (**19**) [22], and sitostenone (**20**) [23] by comparison of their spectroscopic data and specific rotations with those obtained in the literature.

### 2.1. Identification of New Compounds

**Figure 2:**
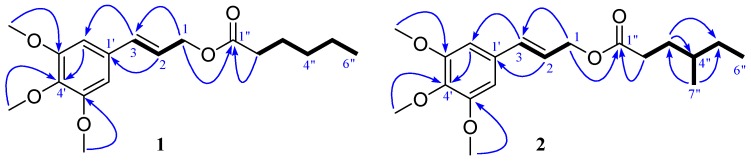
Key ^1^H-^1^H COSY (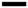) and HMBC (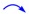) correlations of compounds **1**–**2**.

Compound **1** was obtained as a colorless oil. Its molecular was assigned as C_18_H_26_O_5_ on the basis of positive HRESIMS ([M + Na]^+^ 345.1674, calcd 345.1677) and NMR spectra data (Table 1), which implied six degrees of unsaturation. The IR absorption bands at 1735 cm^−1^ indicated the presence of carbonyl groups. The ^1^H-NMR spectrum of **1** showed three methoxy signals (δ_H_ 3.87 (s, 6H), 3.84 (s, 3H)). The ^13^C- and DEPT-NMR spectra of **1** revealed 18 carbon signals: a carbonyl (δ_C_ 173.8 (C-1”)), a symmetrical benzene (δ_C_ 153.3 (C-3’, 5’), 138.0 (C-4’), 132.0 (C-1’), 103.6 (C-2’, 6’)), a double bond (δ_C_ 134.2 (C-3), 122.9 (C-2)), five methylenes (δ_C_ 64.9 (C-1), 34.3 (C-2”), 31.4 (C-4”), 24.7 (C-3”), 22.4 (C-5”)), and four methyls (δ_C_ 56.1 (C-2*OMe), 61.0 (C-OMe), 14.0 (C-6”)). The NMR data indicated that **1** was a phenylpropanoid, which was very similar with those of **3** [8]. In comparison with **3**, the only difference is a hexanoyl (δ_C_ 173.8 (C-1”), 34.3 (C-2”), 24.7 (C-3”), 31.4 (C-4”), 22.4 (C-5”), 14.0 (C-6”)) in **1** replaced the isovaleryl (δ_C_ 173.0 (C-1”), 43.4 (C-2”), 25.7 (C-3”), 22.4 (C-4”, 5”)) in **3**. The ^1^H-^1^H COSY correlations (Figure 1) between H-2” and H-3”, H-3” and H-4”, H-4” and H-5”, H-5” and H-6”, and the HMBC cross-peaks of H-2” with C-1” confirmed the presence of the hexanoyl in **1**. In the HMBC spectrum, the cross-peak of H-1 with C-1” suggested that the hexanoyl located at C-1 (Figure 2). Hence, the structure of **1** was finally determined as 3’,4’,5’-trimethoxycinnamyl caproate. NMR spectrums show in Supplementary Materials.

Compound **2** was obtained as a colorless oil. The molecular formula of C_19_H_28_O_5_ was determined by HRESIMS ([M + Na]^+^ 359.1842, calcd 359.1834) and NMR spectra data. The NMR data of **2** was closely similar with those of **1**, which suggested that **2** was also a phenylpropanoid. The only difference is that a 4”-methyl-hexanoyl (δ_C_ 174.0 (C-1”), 32.1 (C-2”), 31.4 (C-3”), 34.0 (C-4”), 29.1 (C-5”), 18.8 (C-6”)) in **2** replaced the hexanoyl (δ_C_ 173.8 (C-1”), 34.3 (C-2”), 24.7 (C-3”), 31.4 (C-4”), 22.4 (C-5”), 14.0 (C-6”), 11.4 (C-7”)) in **1**. The ^1^H-^1^H COSY correlations between H-2” and H-3”, H-3” and H-4”, H-4” and H-5”, H-4” and H-7”, H-5” and H-6”, and the HMBC cross-peaks of H-2” with C-1”, confirmed the presence of the 4”-methyl-hexanoyl portion in **2**. In the HMBC spectrum, the cross-peak of H-1 with C-1” suggested that the 4”-methyl-hexanoyl located at C-1 (Figure 2). Thus, the structure of **2** was assigned as 3’,4’,5’-trimethoxycinnamyl-4”-methyl-caproate.

### 2.2. Cytotoxicity Assay

The *in vitro* cytotoxicities of the 90% acetone extract of *S. gaussenii* and compounds **1**–**20** were evaluated against three cancer cell lines, including human cervical carcinoma (HeLa), human gastric carcinoma (BGC-823), and human lung adenocarcinoma (A549). The results indicated that the 90% acetone extract showed cytotoxicity against the A549 cell line (IC_50_ = 0.98 ± 0.1 μg/mL), compound **6** showed cytotoxicities against HeLa (IC_50_ = 0.4 ± 0.1 μM) and BGC-823 (IC_50_ = 0.9 ± 0.2 μM) cancer cell lines, and compound **19** showed cytotoxicities against HeLa (IC_50_ = 1.5 ± 0.4 μM), BGC-823 (IC_50_ = 7.0 ± 0.8 μM) and A549 (IC_50_ = 10.6 ± 1.5 μM) cancer cell lines.

## 3. Materials and Methods

### 3.1. General Experimental Procedures

Spectra were recorded on a Bio-Rad FTS-135 spectrometer (Bio-Rad, Berkeley, CA, USA) with KBr pellets, ν in cm^−1^. UV spectra were measured on SHIMADZU UV-2401PC spectrometer (Shimadzu Corporation, Kyoto, Japan). NMR spectra were conducted on Bruker ARX-600 spectrometers (Bruker Corporation, Rheinstetten, Germany) with TMS as internal standard, chemical shift (δ) was expressed in ppm, and coupling constants (*J*) in Hz. ESI and HR-ESI-MS were taken on an API Qstar-Pulsar-1 mass spectrometer (Thermo Fisher Scientific, Bremen, Germany).

### 3.2. Plant Material

Branches and leaves of *Sabina gaussenii* (Cheng) Cheng et W. T. Wang were collected from Kunming Botany Garden, Yunnan Province, People’s Republic of China, in August 2010. It was identified by Prof. Wei-bang Sun at Kunming Institute of Botany, Chinese Academy of Sciences.

### 3.3. Extraction and Isolation

The powdered air-dried branches and leaves (13 kg) of *S. gaussenii* were extracted with 90% acetone (3 × 40 L) at room temperature and then concentrated under reduced pressure. The concentrated acetone extract (910 g) was dissolved in 60 °C water and partitioned with petroleum ether, EtOAc, and *n*-BuOH, respectively, to afford petroleum ether fraction (170 g), EtOAc fraction (130 g), and *n*-BuOH fraction (250 g).

The petroleum ether fraction (170 g) was separated on an MCI gel column eluted with MeOH–H_2_O (3:7 to 1:0, *v*/*v*) to produce thirteen subfractions A−M. Fraction C (41 g) was separated on a silica gel column and eluted with gradient mixtures of petroleum ether-acetone (from 20:1 to 1:1) and then separated on a column of RP-C_18_ silica gel (MeOH in H_2_O, 60%−80%) to yield five major components, with each purified by semipreparative HPLC (SunFire C18 column, 10 mm × 250 mm, 5 μm, CH_3_CN–H_2_O, 85:15, 3 mL/min) to afford **1** (2.9 mg), **2** (2.3 mg), **3** (16 mg), **10** (11 mg), and **20** (26 mg), respectively. Fraction E was chromatographed on a RP-C_18_ silica gel column (MeOH in H_2_O, 50%−90%) and then purified by semipreparative HPLC with CH_3_CN–H_2_O (80:20, 3 mL/min) as the mobile phase to give compounds **4** (29 mg), **11** (21 mg), **12** (17 mg), **13** (35 mg), **14** (11 mg), **16** (13 mg), and **18** (11 mg), respectively. The EtOAc fraction was subjected to silica gel column (CHCl_3_/MeOH, 9:1 to 7:3) to yield five subfractions N−R. Fraction P was chromatographed on a RP-C_18_ silica gel column (MeOH in H_2_O, 50%−90%) to give **5** (44 mg), **6** (28 mg), **7** (25 mg), **15** (27 mg), and **19** (81 mg), respectively. The *n*-BuOH fraction was subjected to silica gel column (CHCl_3_–MeOH, 10:1 to 0:1), and then subjected to RP-C_18_ column and eluted with MeOH–H_2_O (65:35) to obtain compounds **8** (32 mg), **9** (99 mg), and **17** (28 mg).

### 3.4. Spectroscopic Data

*3’,4’,5’-Trimethoxycinnamyl caproate* (**1**): colorless oil. UV λ_max_ (CH_3_OH) nm (log ε): 270 (4.32), 221 (4.64). IR (KBr) ν_max_ (cm^−1^): 2957, 2935, 1735, 1583, 1507, 1462, 1419, 1242, 1128. ^1^H- and ^13^C-NMR: Table 1. HRESIMS: *m*/*z* 345.1674 (calcd for C_18_H_26_O_5_Na, 345.1677 [M + Na]^+^).

*3’,4’,5’-Trimethoxycinnamyl 4”-methyl-caproate* (**2**): colorless oil. UV λ_max_ (CH_3_OH) nm (logε): 270 (3.52), 220 (3.86). IR (KBr) ν_max_ (cm^−1^): 2959, 2928, 1735, 1584, 1508, 1462, 1420, 1242, 1128. ^1^H- and ^13^C-NMR: Table 1. HRESIMS: *m*/*z* 359.1842 (calcd for C_19_H_28_O_5_Na, 359.1834 [M + Na]^+^).

### 3.5. Bioassay

The cytotoxicities of the 90% acetone extract and compounds (**1**–**20**) against the HeLa, BGC-823, and A549 cancer cell lines were measured using a sulforhodamine B (SRB, Sigma, Saint Louis, MO, USA) assay as described in the literature [24]. Taxol were used as positive controls. Briefly, cells were plated in 96-well culture plates for 24 h and then treated with serial dilutions of all compounds with a maximum concentration of 20 μg/mL. After being incubated for 48 h under a humidified atmosphere of 5% CO_2_ at 37 °C, cells were fixed with 25 μL of ice-cold 50% trichloroacetic acid and incubated at 4 °C for 1 h. After washing with distilled water and air-drying, the plate was stained for 15 min with 100 μL of 0.4% SRB in 1% glacial acetic acid. The plates were washed with 1% acetic acid and air-dried. For reading the plate, the protein-bound dye was dissolved in 100 μL of 10 mM Tris base. The absorbance was measured at 560 nm on a microplate spectrophotometer (Molecular Devices SpectraMax 340, MWG-Biotech, Inc., Sunnyvale, CA, USA). All tests were performed in triplicate, and results are expressed as IC_50_ values.

## 4. Conclusions

This work was part of a series of investigations on anti-tumor compounds from Gymnospermae plants. Compounds **1**–**2** were found to be new cinnamyl isovalerate derivatives, and the other eighteen compounds were found for the first time from *S. gaussenii*. The 90% acetone extract showed significant cytotoxicity against the A549 cell line (IC_50_ = 0.98 ± 0.1 μg/mL). The next bioassay guided isolation led to the discovery of two cytotoxic compounds, compound **6** showed cytotoxicities against HeLa (IC_50_ = 0.4 ± 0.1 μM) and BGC-823 (IC_50_ = 0.9 ± 0.2 μM) cancer cell lines, and compound **19** showed cytotoxicities against HeLa (IC_50_ = 1.5 ± 0.4 μM), BGC-823 (IC_50_ = 7.0 ± 0.8 μM), and A549 (IC_50_ = 10.6 ± 1.5 μM) cancer cell lines. The result indicated that the podophyllotoxin type and the diterpene type compounds were the major cytotoxic constituents in this species, which might be worthy of more extensive investigation so that more novel bioactive compounds can be discovered in the future.

## Figures and Tables

**Table 1:** ^1^H (600 MHz) and ^13^C (150 MHz) NMR spectroscopic data of **1**–**2** in CDCl_3_. (*J* in Hz, δ in ppm).

No.	1		2	
	δ_H_	δ_C_	δ_H_	δ_C_
1	4.72 (dd, 6.5, 1.0, 2H)	64.9	4.72 (dd, 6.5, 1.0, 2H)	64.9
2	6.21 (dt, 15.7, 6.5, 1H)	122.9	6.21 (dt, 15.7, 6.5, 1H)	122.8
3	6.57 (d, 15.7, 1H)	134.2	6.58 (d, 15.7, 1H)	134.2
1’		132.0		131.9
2’	6.61 (s, 1H)	103.6	6.61 (s, 1H)	103.5
3’		153.3		153.3
4’		138.0		138.0
5’		153.3		153.3
6’	6.61 (s, 1H)	103.5	6.61 (s, 1H)	103.5
3’-OMe	3.87 (s, 3H)	56.1	3.87 (s, 3H)	56.0
4’-OMe	3.84 (s, 3H)	61.0	3.84 (s, 3H)	60.9
5’-OMe	3.87 (s, 3H)	56.1	3.87 (s, 3H)	56.0
1”		173.8		174.0
2”	2.35 (t, 7.6 Hz, 2H)	34.3	2.35 (m, 2H)	32.1
3”	1.65 (m, 2H)	24.7	1.70 (m, 1H) 1.46 (m, 1H)	31.4
4”	1.31 (m, 2H)	31.4	1.34 (m, 1H)	34.0
5”	1.31 (m, 2H)	22.4	1.34 (m, 1H) 1.16 (m, 1H)	29.1
6”	0.89 (t, 7.0 Hz, 3H)	14.0	0.87 (m, 3H)	18.8
7”			0.87 (m, 3H)	11.3

## Supplementary Materials

The ^1^H- and ^13^C-NMR data of **1**–**20**, HR-ESI-MS, 2D-NMR spectra of compounds **1**–**2** can be accessed at: http://www.mdpi.com/1420-3049/21/5/571/s1.
