# Supplementary material for: Two New Cinnamyl Isovalerate Derivatives from Sabina gaussenii
Source: Molecules. 2016 Apr 29;21(5):571. doi: 10.3390/molecules21050571 (PMC6273497; doi:10.3390/molecules21050571)
Supplement: Supplementary file 1 [file molecules-21-00571-s001.pdf]

Supplementary Materials: Two New Cinnamyl Isovalerate Derivatives from *Sabina gaussenii*

Zhang-Hua Sun, Ning-Hua Tan, Guang-Zhi Zeng and Yu-Mei Zhang

1. Contents:

Figure S1 ^{1}H-NMR spectrum of 3′,4′,5′-trimethoxycinnamyl caproate (**1**) in $CDCl_3$.
Figure S2 ^{13}C-NMR and DEPT spectra of 3,4,5-trimethoxycinnamyl caproate (**1**) in $CDCl_3$.
Figure S3 HSQC spectrum of 3,4,5-trimethoxycinnamyl caproate (**1**) in $CDCl_3$.
Figure S4 ^{1}H-^{1}H COSY spectrum of 3,4,5-trimethoxycinnamyl caproate (**1**) in $CDCl_3$.
Figure S5 HMBC spectrum of 3,4,5-trimethoxycinnamyl caproate (**1**) in $CDCl_3$.
Figure S6 HR-ESI-MS spectrum of 3′,4′,5′-trimethoxycinnamyl caproate (**1**) in $CDCl_3$.
Figure S7 ^{1}H-NMR spectrum of 3,4,5-trimethoxycinnamyl-4″-methyl-caproate (**2**) in $CDCl_3$.
Figure S8 ^{13}C-NMR and DEPT spectra of 3,4,5-trimethoxycinnamyl-4″-methyl -caproate (**2**) in $CDCl_3$.
Figure S9 HSQC spectrum of 3,4,5-trimethoxycinnamyl-4″-methyl-caproate (**2**) in $CDCl_3$.
Figure S10 ^{1}H-^{1}H COSY spectrum of 3,4,5-trimethoxycinnamyl-4″-methyl-caproate (**2**) in $CDCl_3$.
Figure S11 HMBC spectrum of 3,4,5-trimethoxycinnamyl-4″-methyl-caproate (**2**) in $CDCl_3$.
Figure S12 HR-ESI-MS spectrum of 3,4,5-trimethoxycinnamyl-4″-methyl-caproate (**2**) in $CDCl_3$.
Figure S13 ^{1}H-NMR spectrum of 3,4,5-dimethoxycinnamyl isovalerate (**3**) in $CDCl_3$.
Figure S14 ^{13}C-NMR spectrum of 3,4,5-dimethoxycinnamyl isovalerate (**3**) in $CDCl_3$.
Figure S15 ^{1}H-NMR spectrum of 3,4,5-dimethoxycinnamyl alcohol (**4**) in $CDCl_3$.
Figure S16 ^{13}C-NMR spectrum of 3,4,5-dimethoxycinnamyl alcohol (**4**) in $CDCl_3$.
Figure S17 ^{1}H-NMR spectrum of dihydrosesamin (**5**) in $CDCl_3$.
Figure S18 ^{13}C-NMR spectrum of dihydrosesamin (**5**) in $CDCl_3$.
Figure S19 ^{1}H-NMR spectrum of 4′-*O*-demethylepipodophyllotoxin (**6**) in $CDCl_3$.
Figure S20 ^{13}C-NMR spectrum of 4′-*O*-demethylepipodophyllotoxin (**6**) in $CDCl_3$.
Figure S21 ^{1}H-NMR spectrum of 7-hydroxy coumarin (**7**) in $CDCl_3$ and acetone-d_6 (1:1).
Figure S22 ^{13}C-NMR spectrum of 7-hydroxy coumarin (**7**) in $CDCl_3$ and acetone-d_6 (1:1).
Figure S23 ^{1}H-NMR spectrum of 7-β-D-glucosyloxy coumarin (**8**) in CD_3OD.
Figure S24 ^{13}C-NMR spectrum of 7-β-D-glucosyloxy coumarin (**8**) in CD_3OD.
Figure S25 ^{1}H-NMR spectrum of 1-β-D-glucosyloxy-2-(3,4-methylenedioxyphenyl) -propane -l,3-diol (**9**) in CD_3OD.
Figure S26 ^{13}C-NMR spectrum of 1-β-D-glucosyloxy-2-(3,4-methylenedioxyphenyl) -propane -l,3-diol (**9**) in CD_3OD.
Figure S27 ^{1}H-NMR spectrum of lβ,6α-dihydroxy-4(14)-eudesmene (**10**) in $CDCl_3$.
Figure S28 ^{13}C-NMR spectrum of lβ,6α-dihydroxy-4(14)-eudesmene (**10**) in $CDCl_3$.
Figure S29 ^{1}H-NMR spectrum of selin-4(15)-en-1β, 11-diol (**11**) in $CDCl_3$.
Figure S30 ^{13}C-NMR spectrum of selin-4(15)-en-1β, 11-diol (**11**) in $CDCl_3$.
Figure S31 ^{1}H-NMR spectrum of 4-eudesmene-1β,11-diol (**12**) in $CDCl_3$.
Figure S32 ^{13}C-NMR spectrum of 4-eudesmene-1β,11-diol (**12**) in $CDCl_3$.
Figure S33 ^{1}H-NMR spectrum of 7-*epi*-4-eudesmene-1β,11-diol (**13**) in $CDCl_3$.
Figure S34 ^{13}C-NMR spectrum of 7-*epi*-4-eudesmene-1β,11-diol (**13**) in $CDCl_3$.
Figure S35 ^{1}H-NMR spectrum of 3-eudesmene-1β,11-diol (**14**) in $CDCl_3$.
Figure S36 ^{13}C-NMR spectrum of 3-eudesmene-1β,11-diol (**14**) in $CDCl_3$.
Figure S37 ^{1}H-NMR spectrum of 8α,11-elemodiol (**15**) in acetone-d_6.
Figure S38 ^{13}C-NMR spectrum of 8α,11-elemodiol (**15**) in acetone-d_6.
Figure S39 ^{1}H-NMR spectrum of hinokiic acid (**16**) in $CDCl_3$.
Figure S40 ^{13}C-NMR spectrum of hinokiic acid (**16**) in $CDCl_3$.
Figure S41 ^{1}H-NMR spectrum of corchoionoside C (**17**) in CD_3OD.
Figure S42 ^{13}C-NMR spectrum of corchoionoside C (**17**) in CD_3OD.

Figure S43 ^{1}H-NMR spectrum of hinokiol (**18**) in pyridine-*d*6.
Figure S44 ^{13}C-NMR spectrum of hinokiol (**18**) in pyridine-*d*6.
Figure S45 ^{1}H-NMR spectrum of isocupressic acid (**19**) in $CDCl_3$.
Figure S46 ^{13}C-NMR spectrum of isocupressic acid (**19**) in $CDCl_3$.
Figure S47 ^{1}H-NMR spectrum of sitostenone (**20**) in $CDCl_3$.
Figure S48 ^{13}C-NMR spectrum of sitostenone (**20**) in $CDCl_3$.

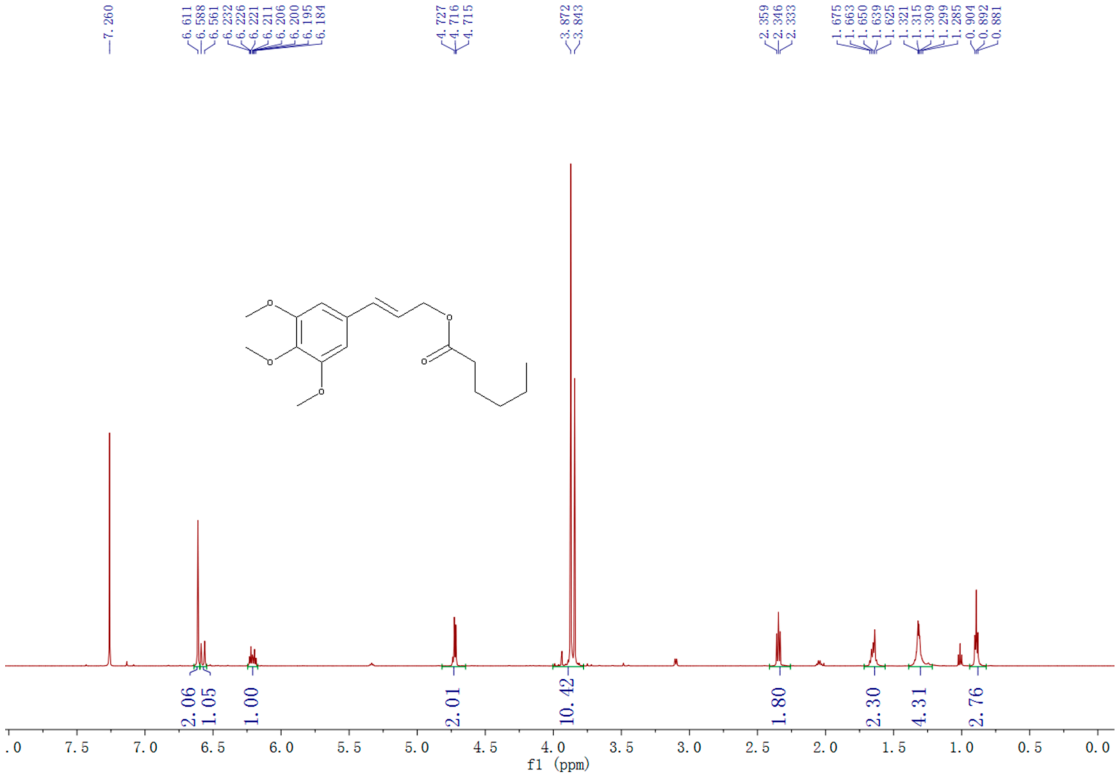

Figure S1. ^{1}H-NMR spectrum of 3,4,5-trimethoxycinnamyl caproate (**1**) in $CDCl_3$.

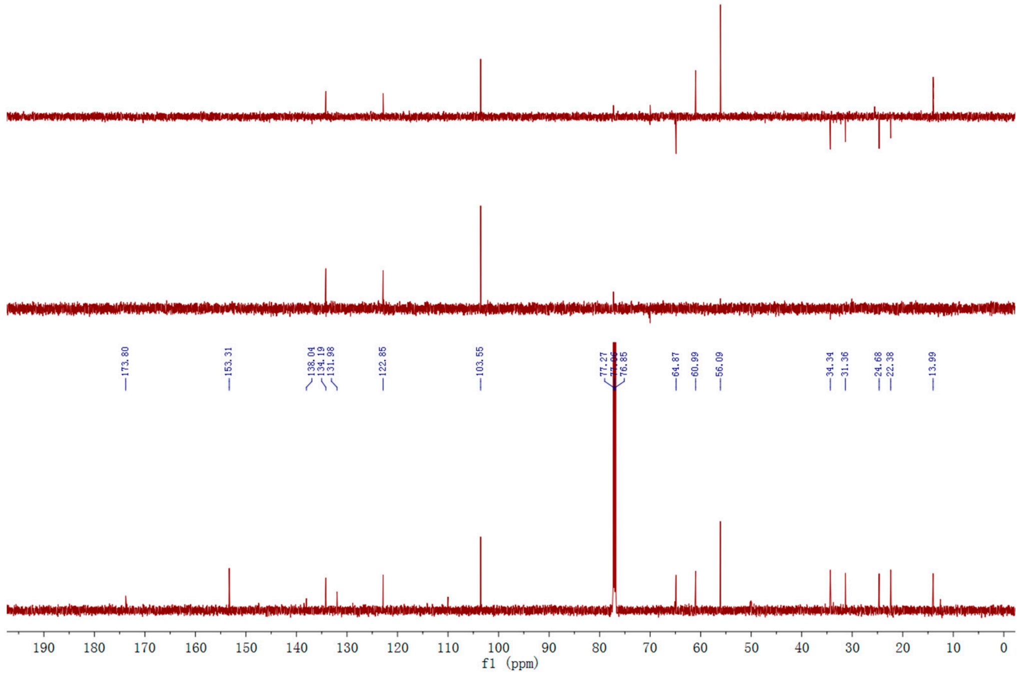

Figure S2. ^{13}C-NMR and DEPT spectra of 3,4,5-trimethoxycinnamyl caproate (**1**) in $CDCl_3$.

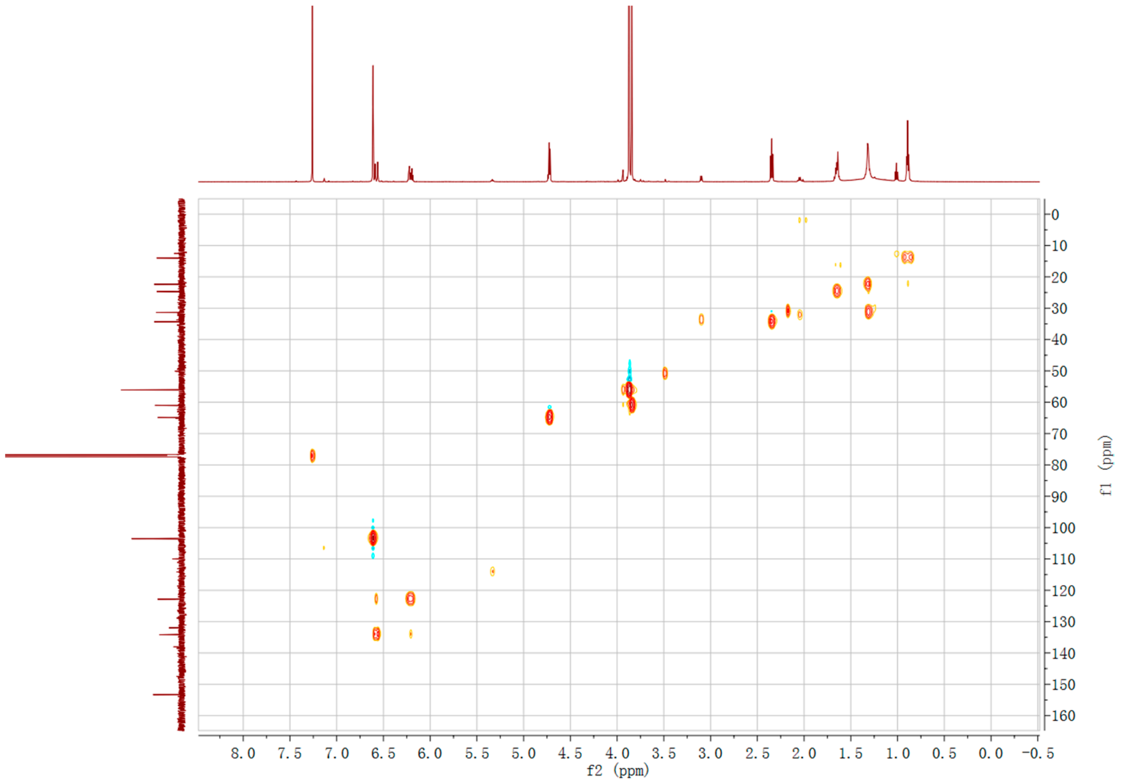

Figure S3. HSQC spectrum of 3,4,5-trimethoxycinnamyl caproate (**1**) in $CDCl_3$.

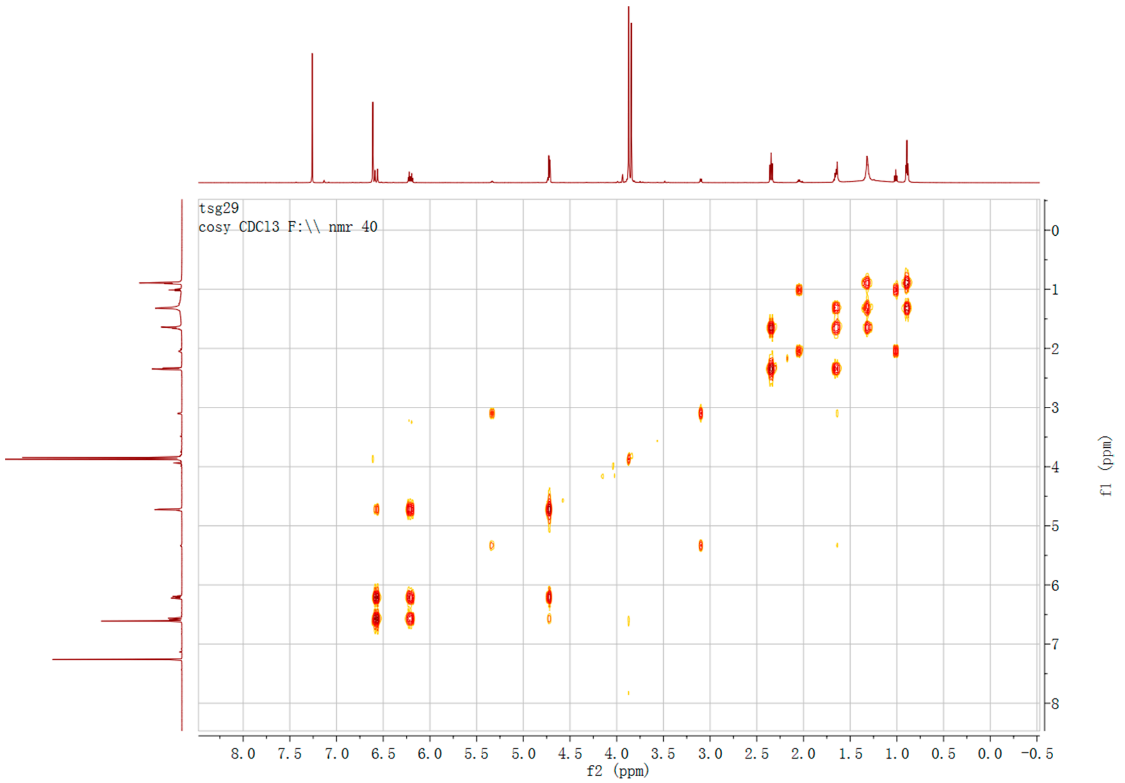

Figure S4. ^1H-^1H COSY spectrum of **1** 3,4,5-trimethoxycinnamyl caproate (**1**) in $CDCl_3$.

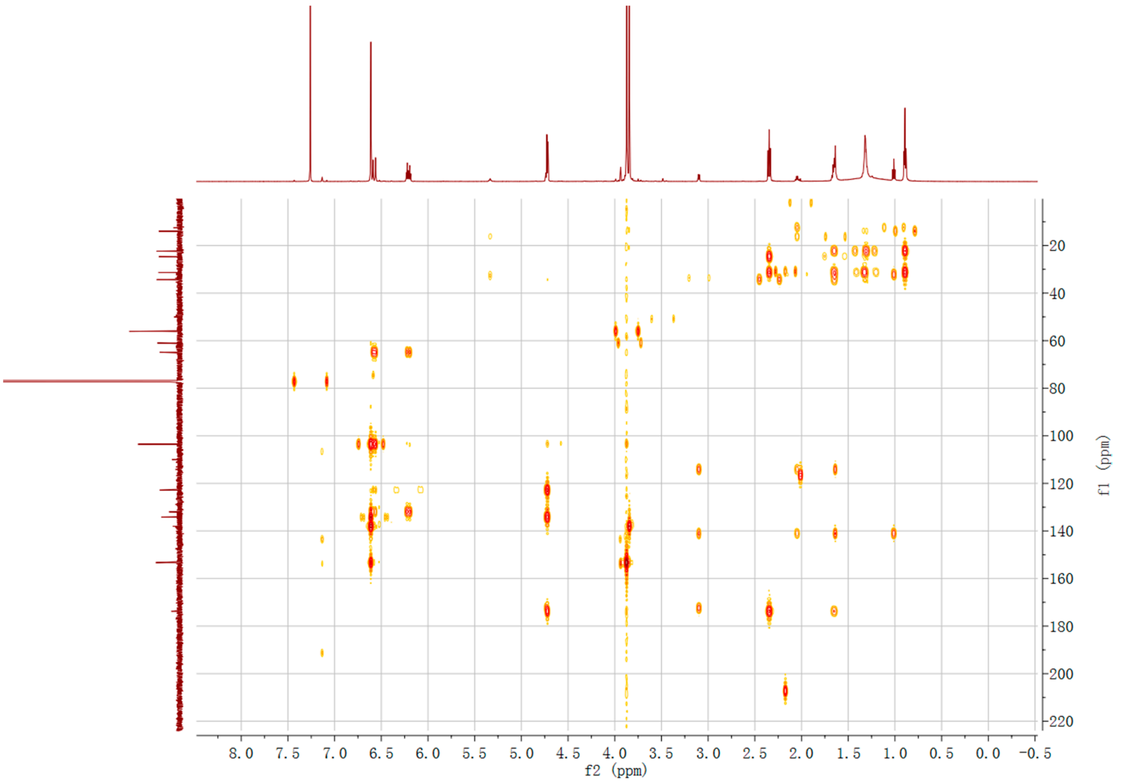

Figure S5. HMBC spectrum of 3′,4′,5′-trimethoxycinnamyl caproate (**1**) in $CDCl_3$.

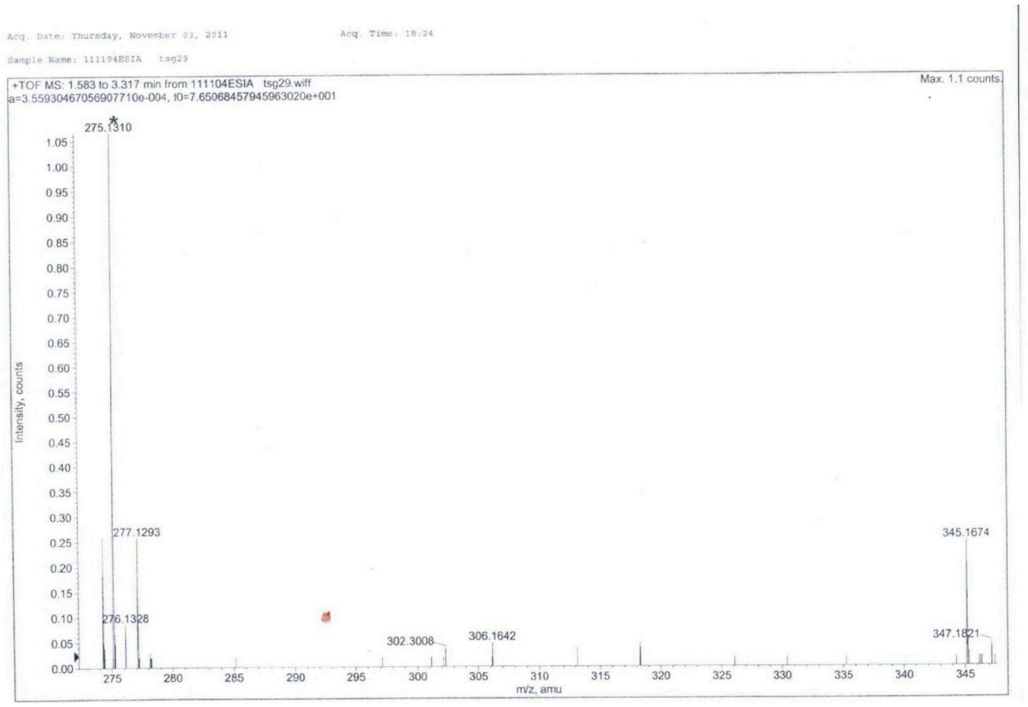

Figure S6. HR-ESI-MS spectrum of 3′,4′,5′-trimethoxycinnamyl caproate (1) in $CDCl_3$.

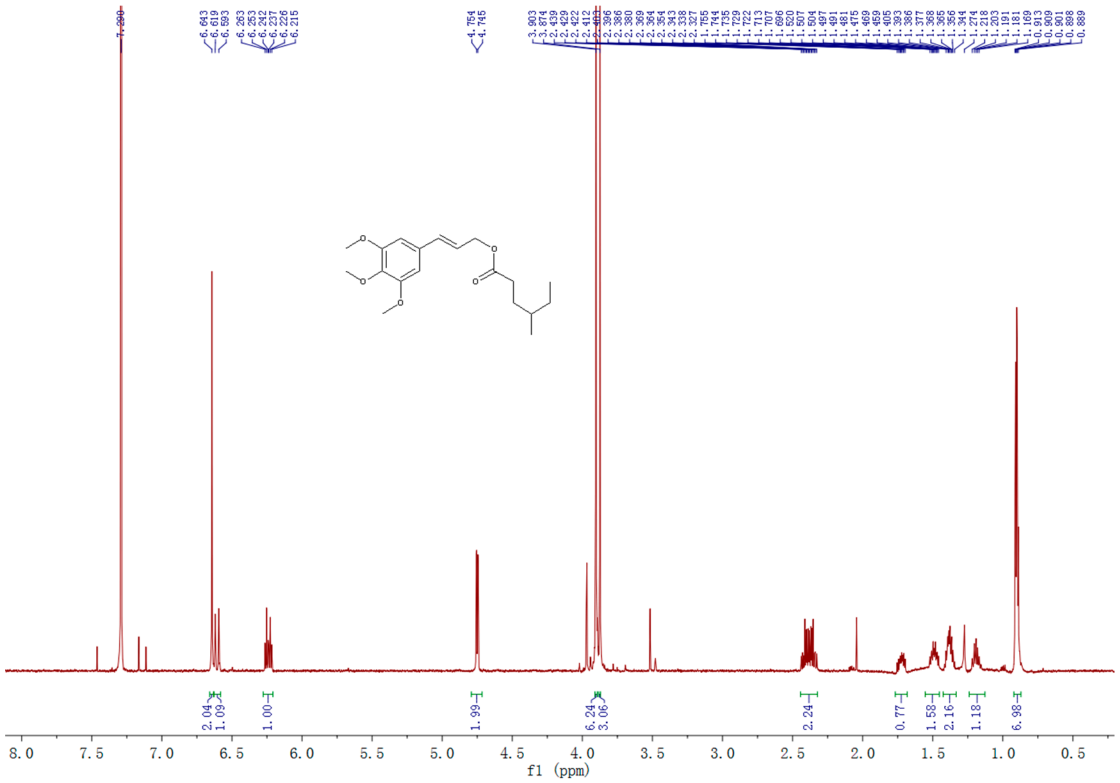

Figure S7. ^{1}H-NMR spectrum of 3,4,5-trimethoxycinnamyl-4′′-methyl-caproate (**2**) in $CDCl_3$.

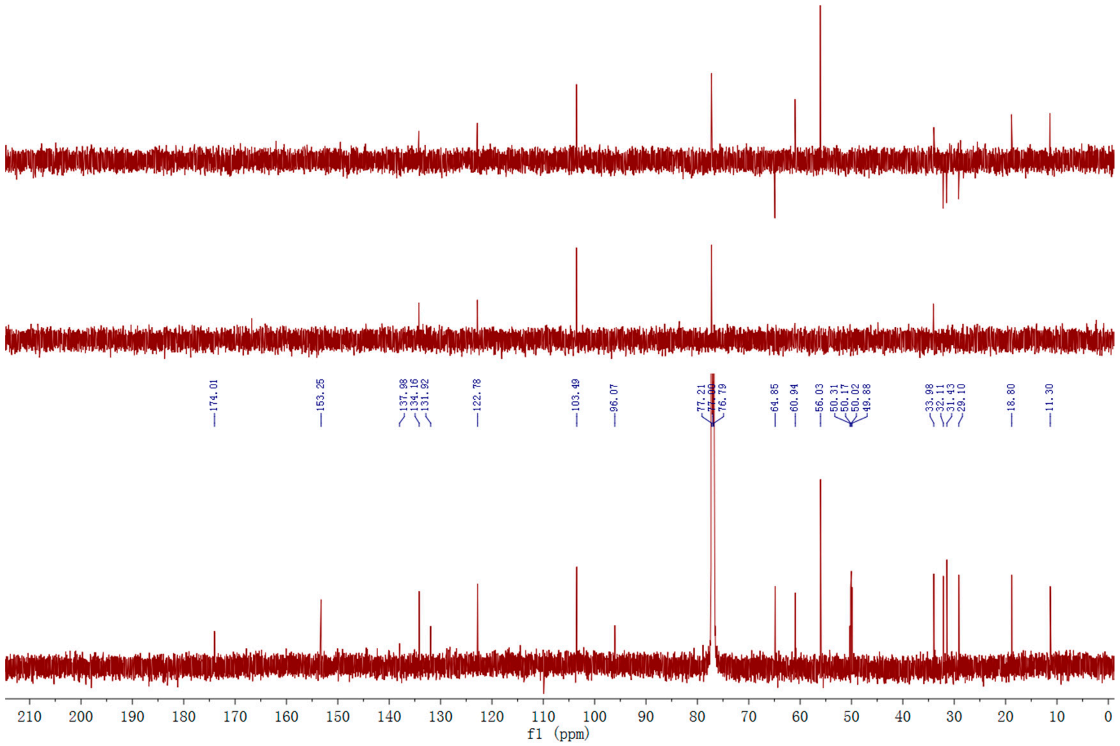

Figure S8. ^{13}C-NMR and DEPT spectrum of 3,4,5-trimethoxycinnamyl-4′′-methyl-caproate (**2**) in $CDCl_3$..

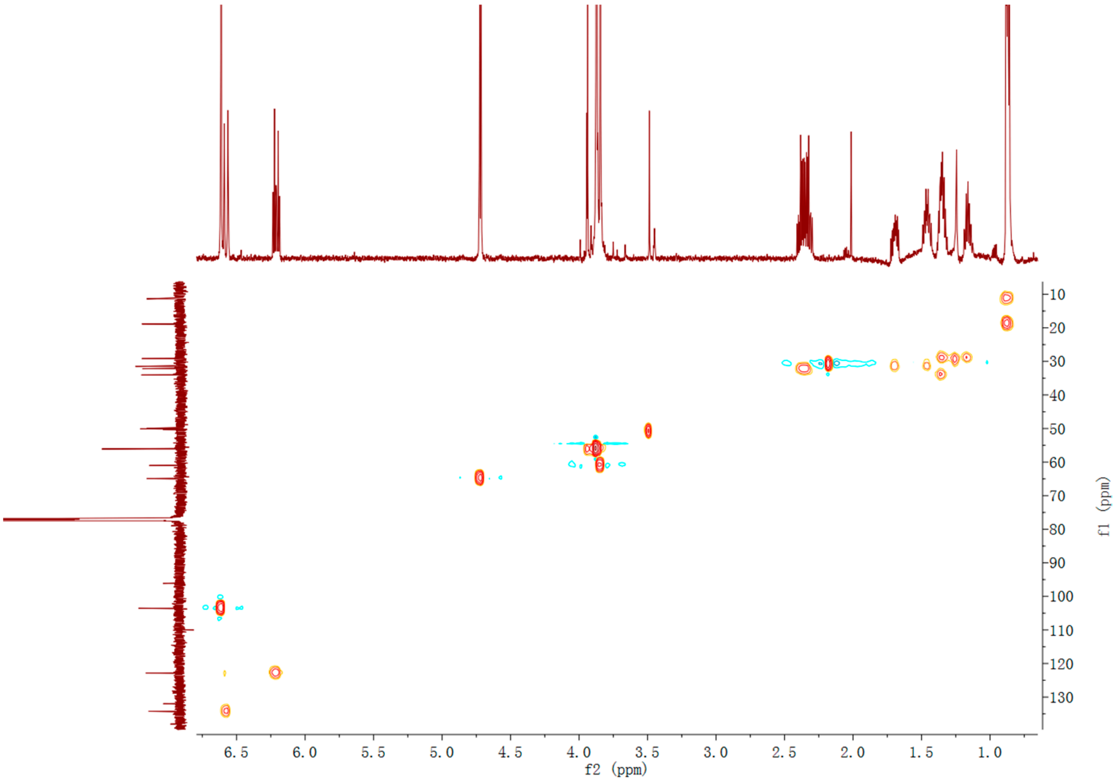

Figure S9. HSQC spectrum of 3,4,5-trimethoxycinnamyl-4′′-methyl-caproate (**2**) in $CDCl_3$.

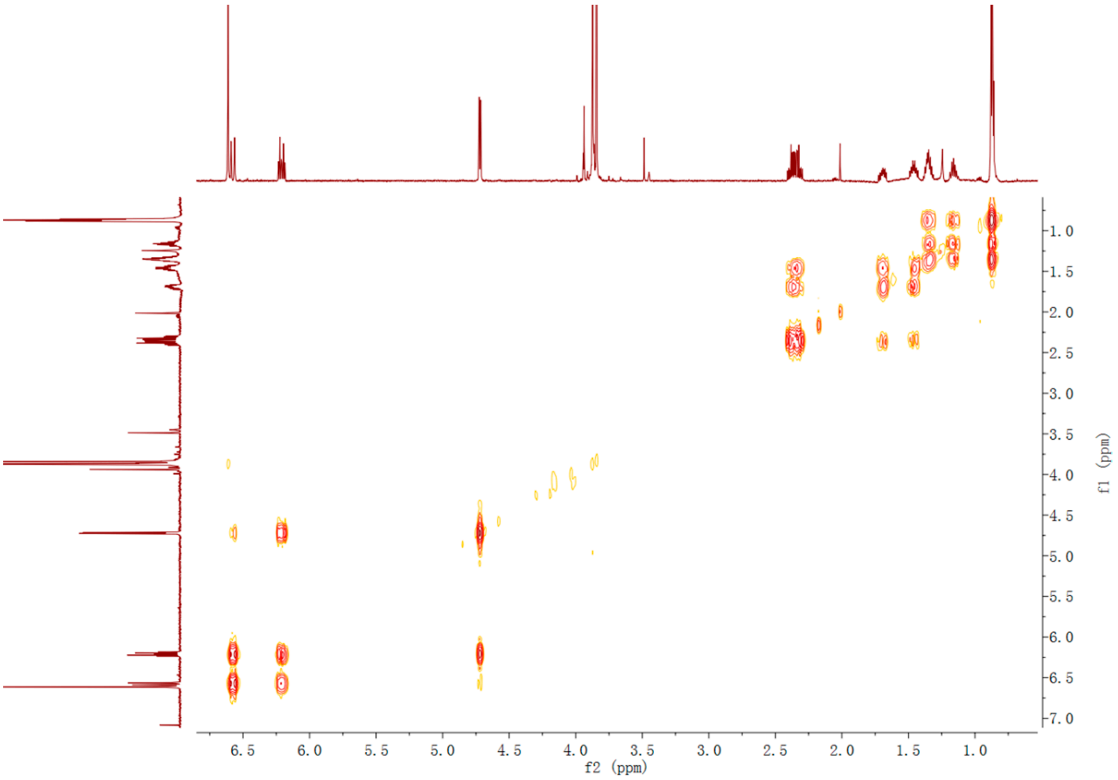

Figure S10. ^{1}H-^{1}H COSY spectrum of 3,4,5-trimethoxycinnamyl-4′′-methyl-caproate (**2**) in $CDCl_3$.

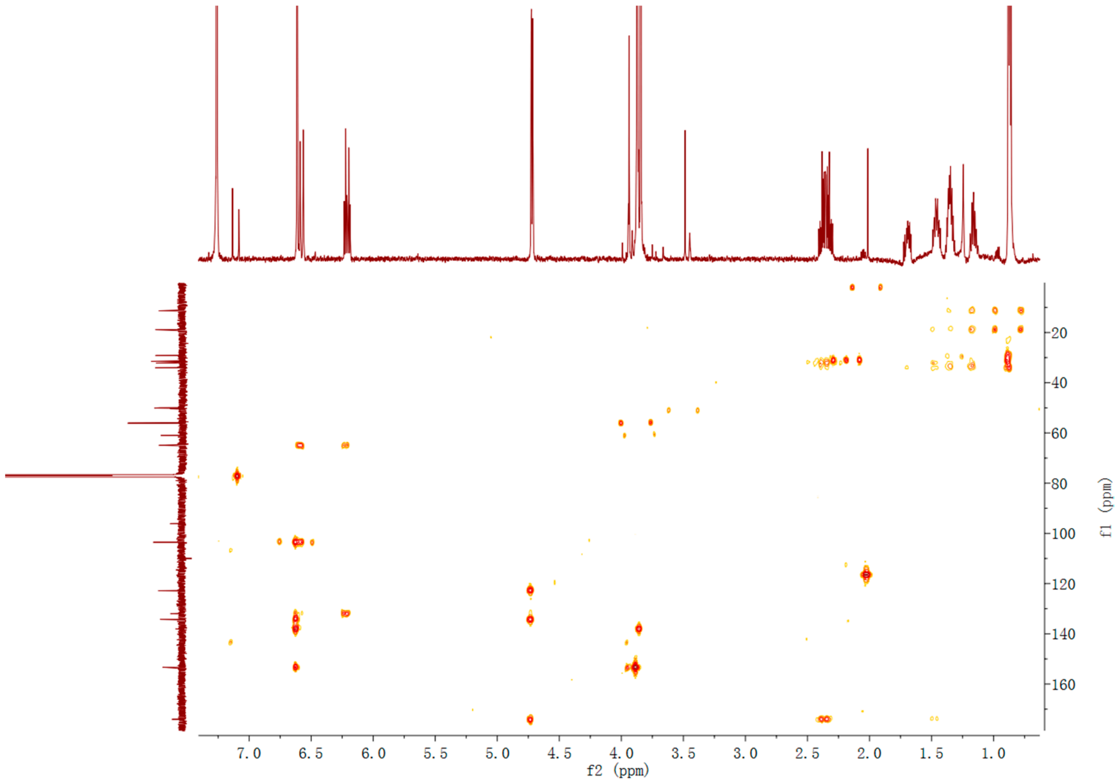

Figure S11. HMBC spectrum of 3,4,5-trimethoxycinnamyl-4′′-methyl-caproate (**2**) in $CDCl_3$.

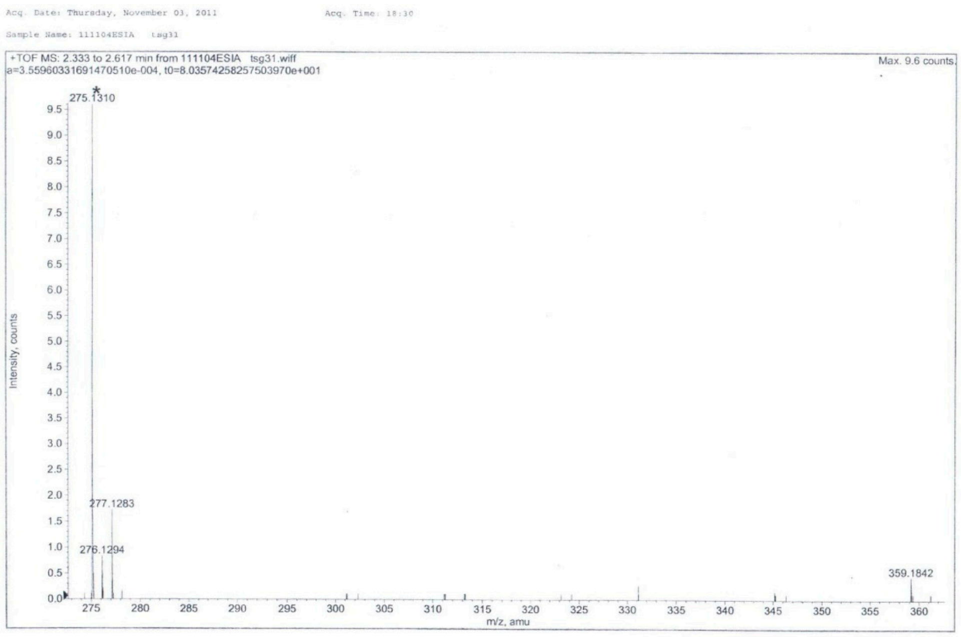

Figure S12. HR-ESI-MS spectrum of 3,4,5-trimethoxycinnamyl-4′′-methyl-caproate (**2**) in $CDCl_3$.

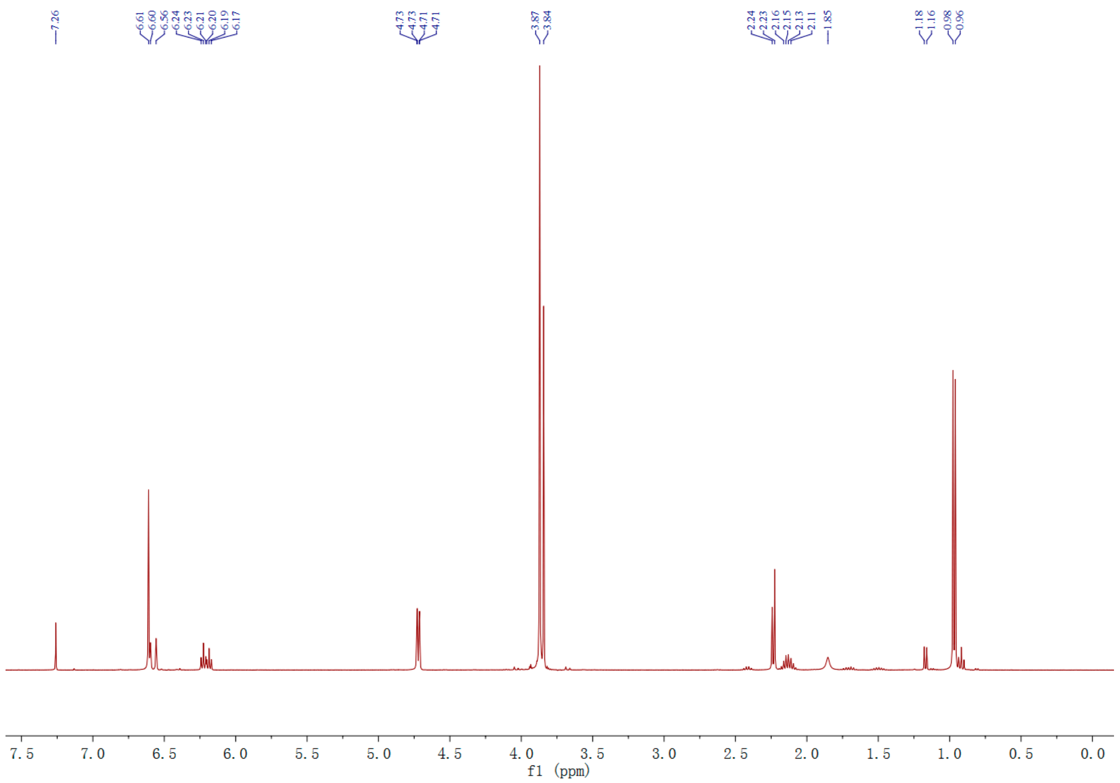

Figure S13. ^{1}H-NMR spectrum of 3,4,5-dimethoxycinnamyl isovalerate (**3**) in $CDCl_3$.

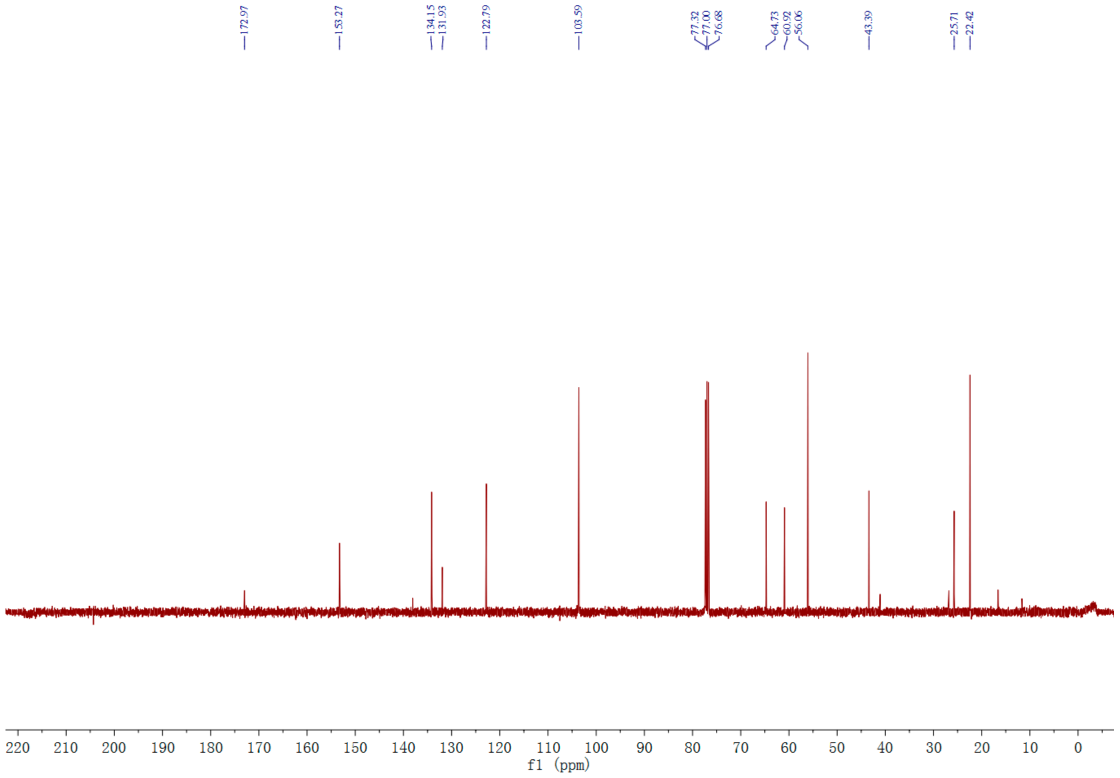

Figure S14 ^{13}C-NMR spectrum of 3,4,5-dimethoxycinnamyl isovalerate (**3**) in $CDCl_3$.

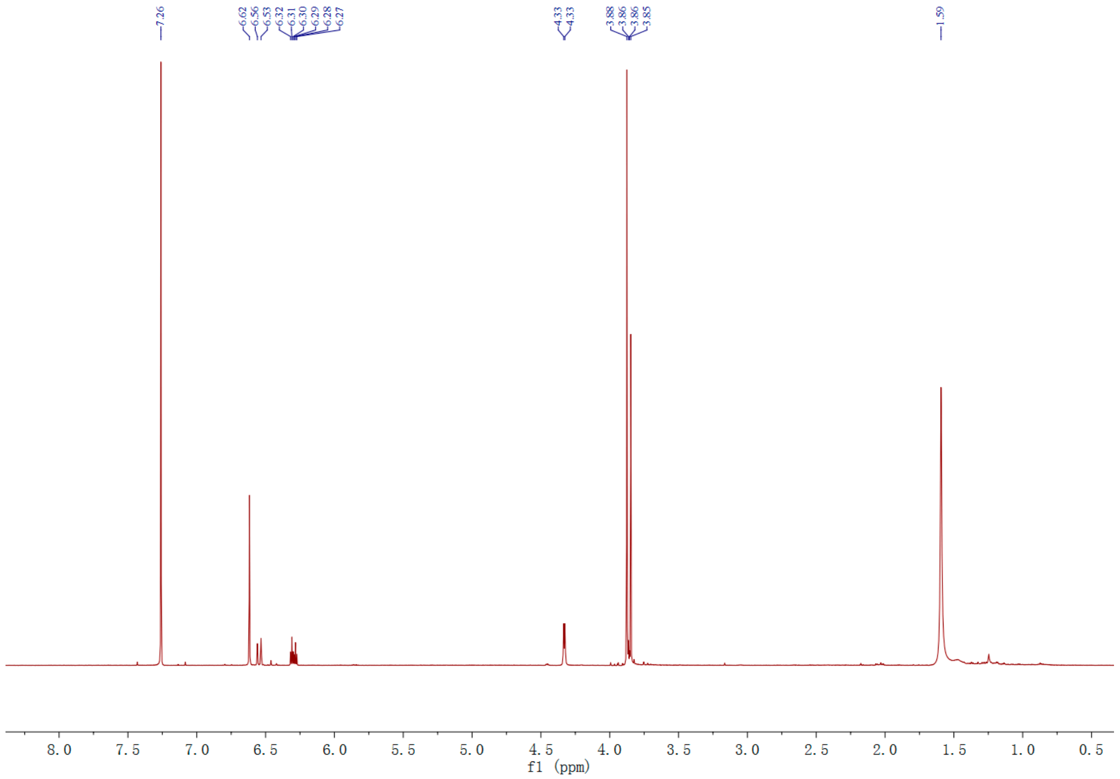

Figure S15. ^{1}H-NMR spectrum of 3,4,5-dimethoxycinnamyl alcohol (**4**) in $CDCl_3$.

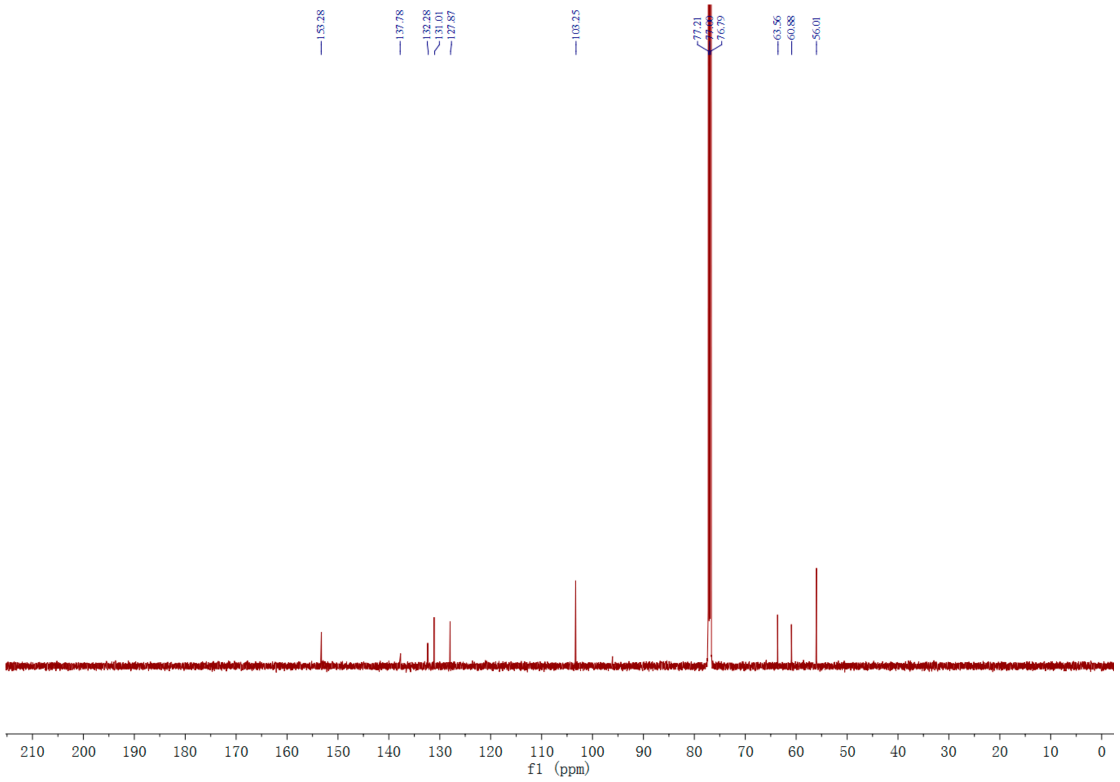

Figure S16. ^{13}C-NMR spectrum of 3,4,5-dimethoxycinnamyl alcohol (**4**) in $CDCl_3$.

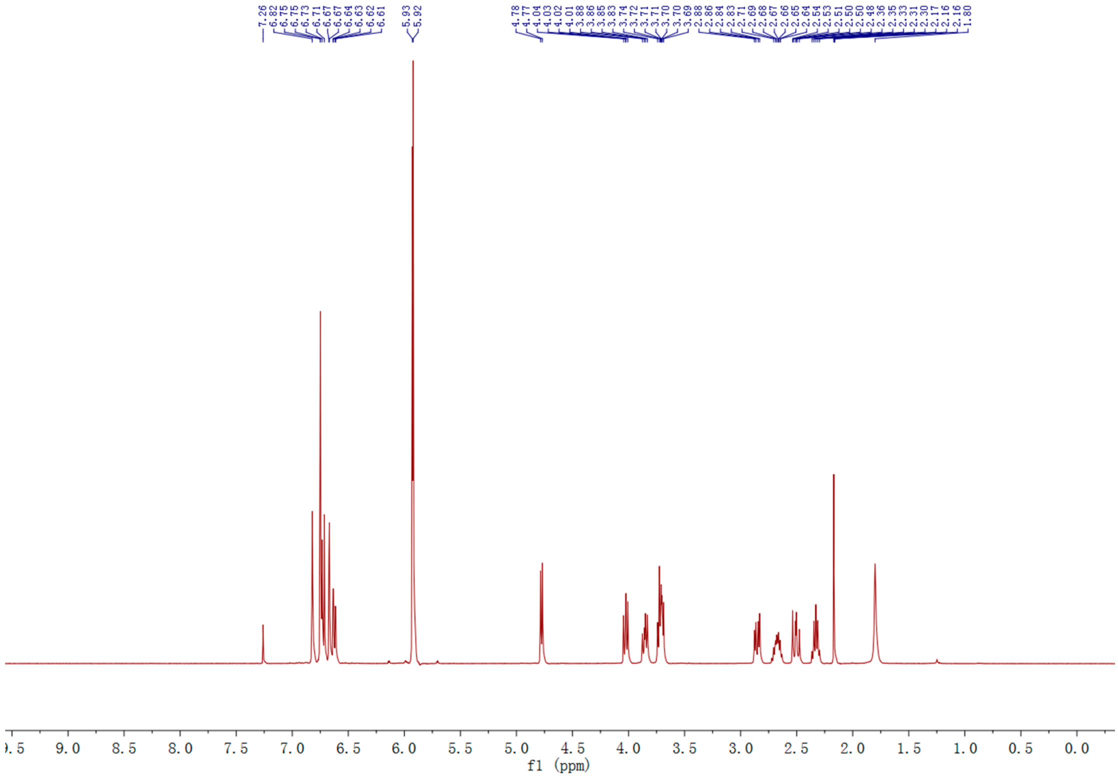

Figure S17. ^{1}H-NMR spectrum of dihydrosesamin (**5**) in CDCl3.

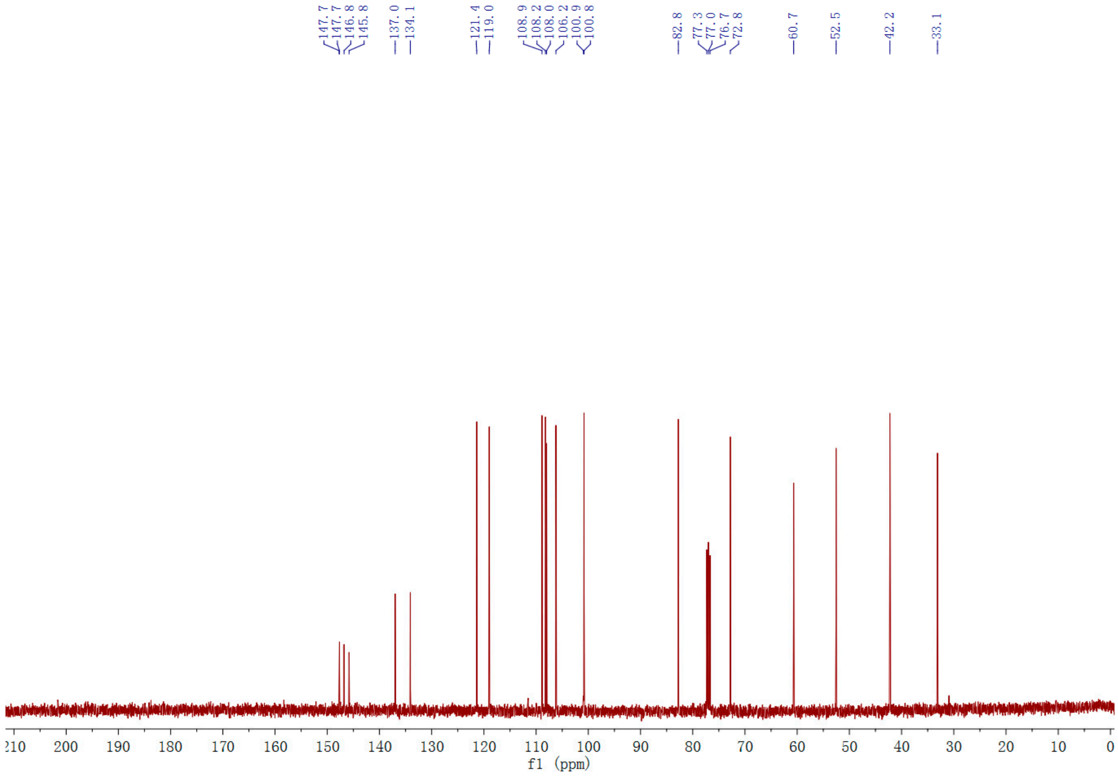

Figure S18. ^{13}C-NMR spectrum of dihydrosesamin (**5**) in $CDCl_3$.

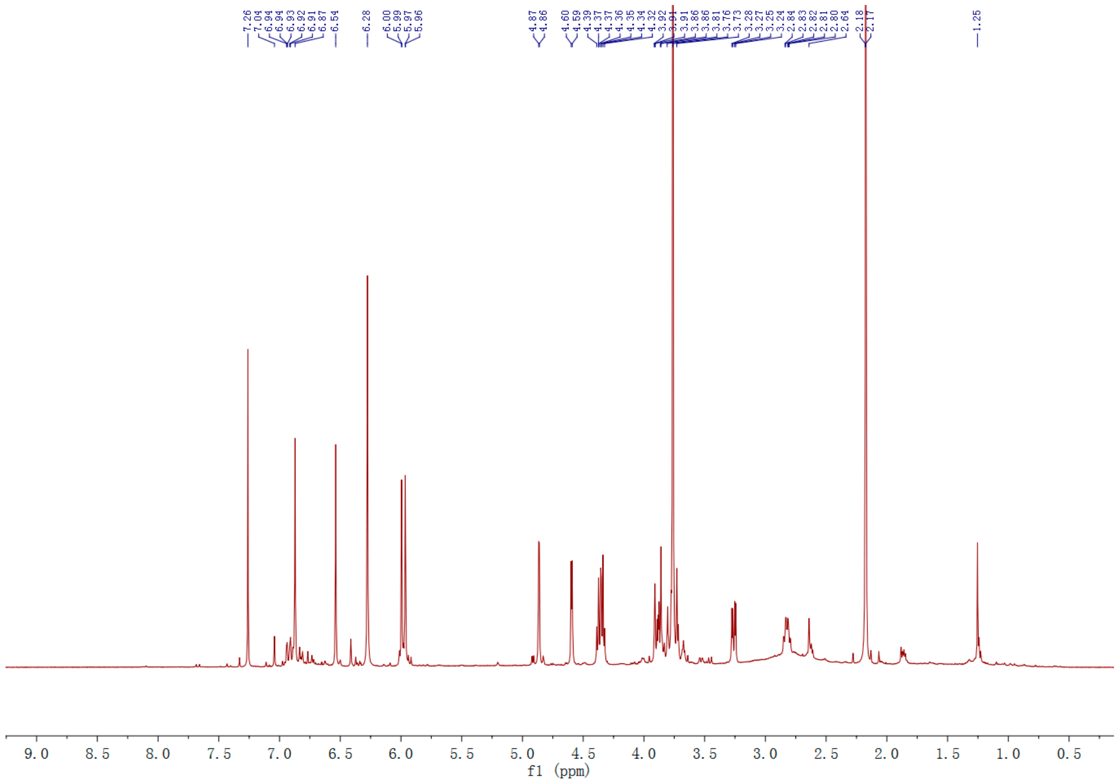

Figure S19. ^{1}H-NMR spectrum of 4′-*O*-demethylepipodophyllotoxin (**6**) in $CDCl_3$.

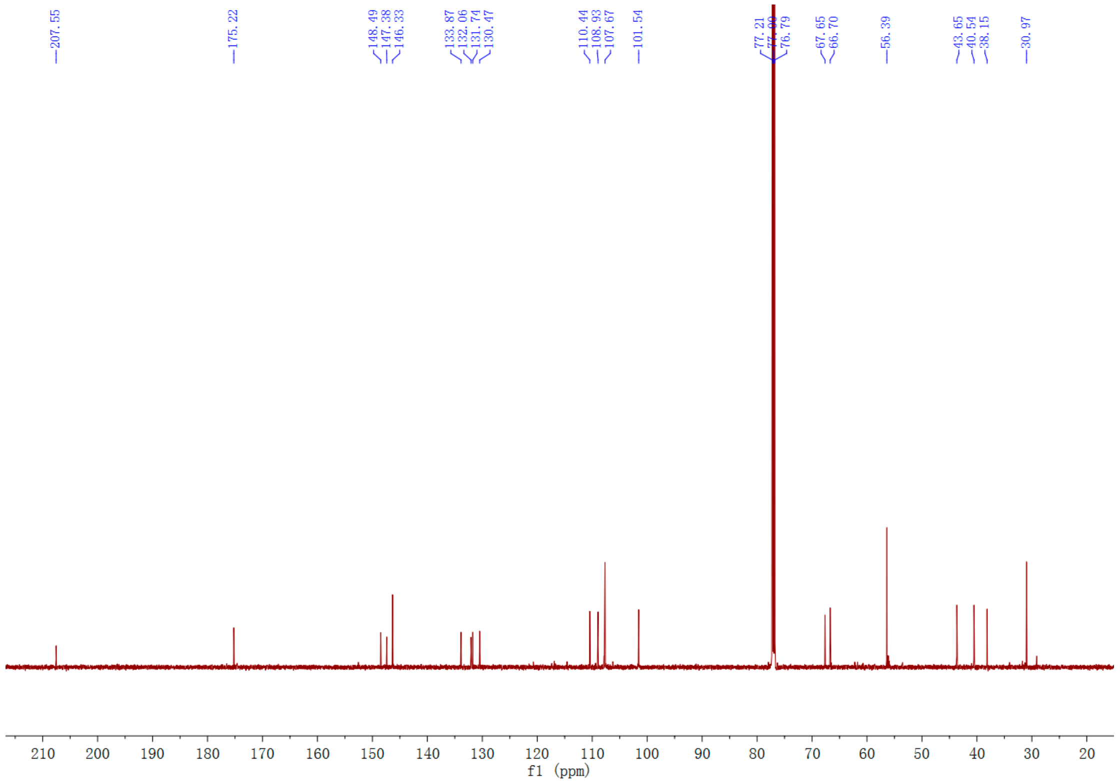

Figure S20. ^{13}C-NMR spectrum of 4′-*O*-demethylepipodophyllotoxin (**6**) in $CDCl_3$.

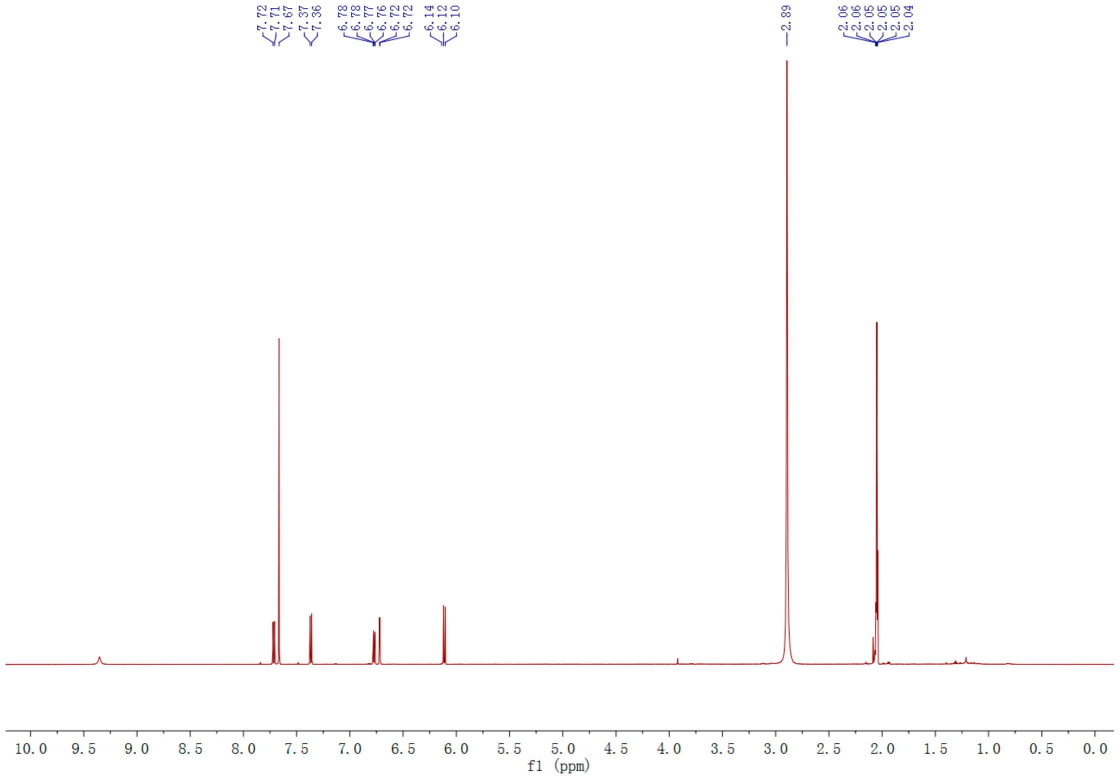

Figure S21. ^{1}H-NMR spectrum of 7-hydroxy coumarin (**7**) in $CDCl_3$. and acetone-d_6 (1:1).

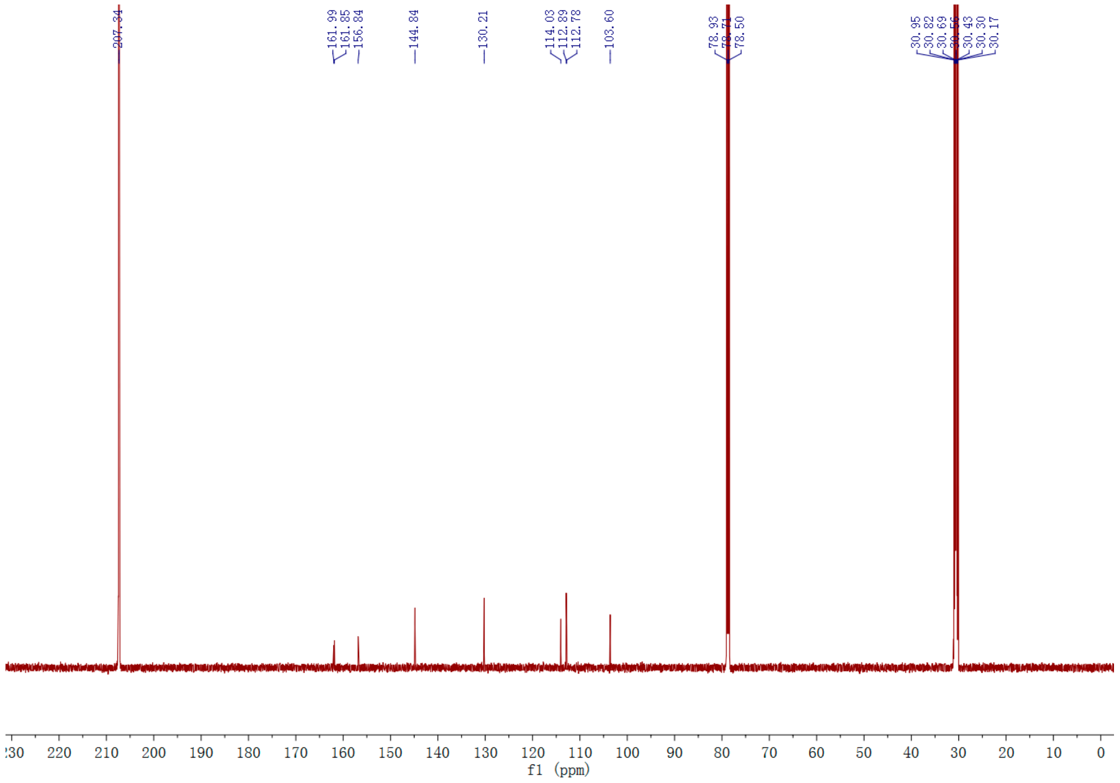

Figure S22. ^{13}C-NMR spectrum of 7-hydroxy coumarin (**7**) in $CDCl_3$. and acetone-d_6 (1:1).

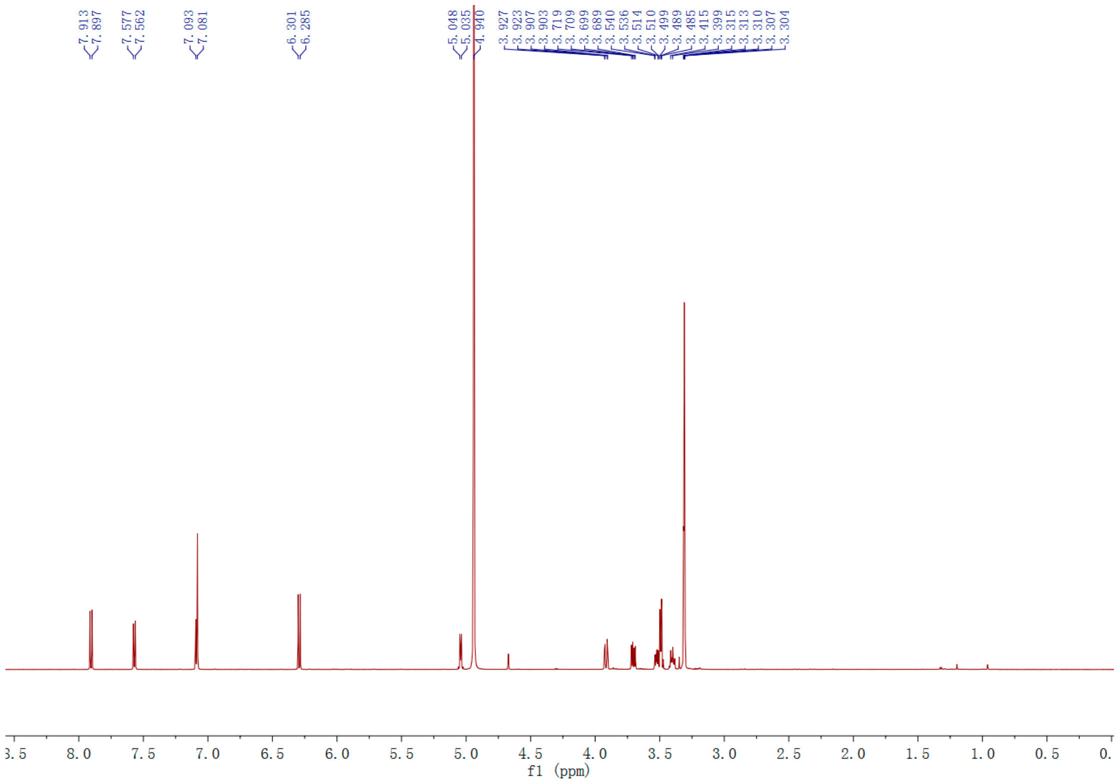

Figure S23. ^{1}H-NMR spectrum of 7-β-D-glucosyloxy coumarin (**8**) in CD_3OD.

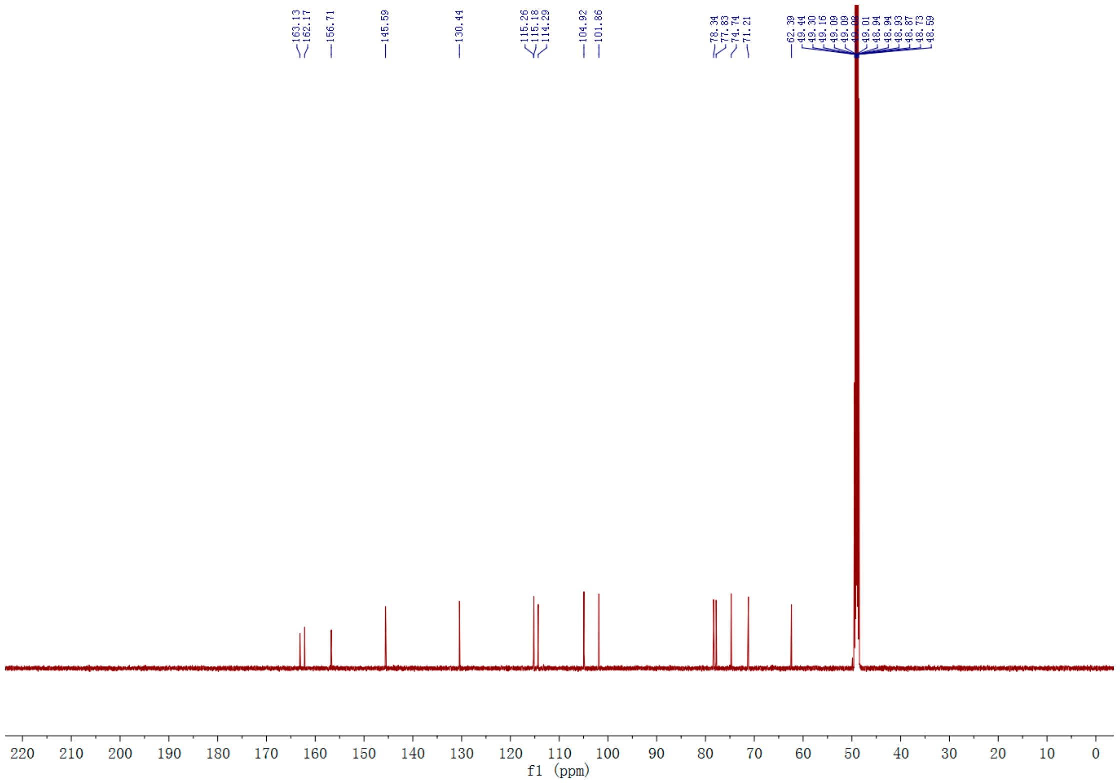

Figure S24 ^{13}C-NMR spectrum of 7-β-D-glucosyloxy coumarin (**8**) in CD_3OD.

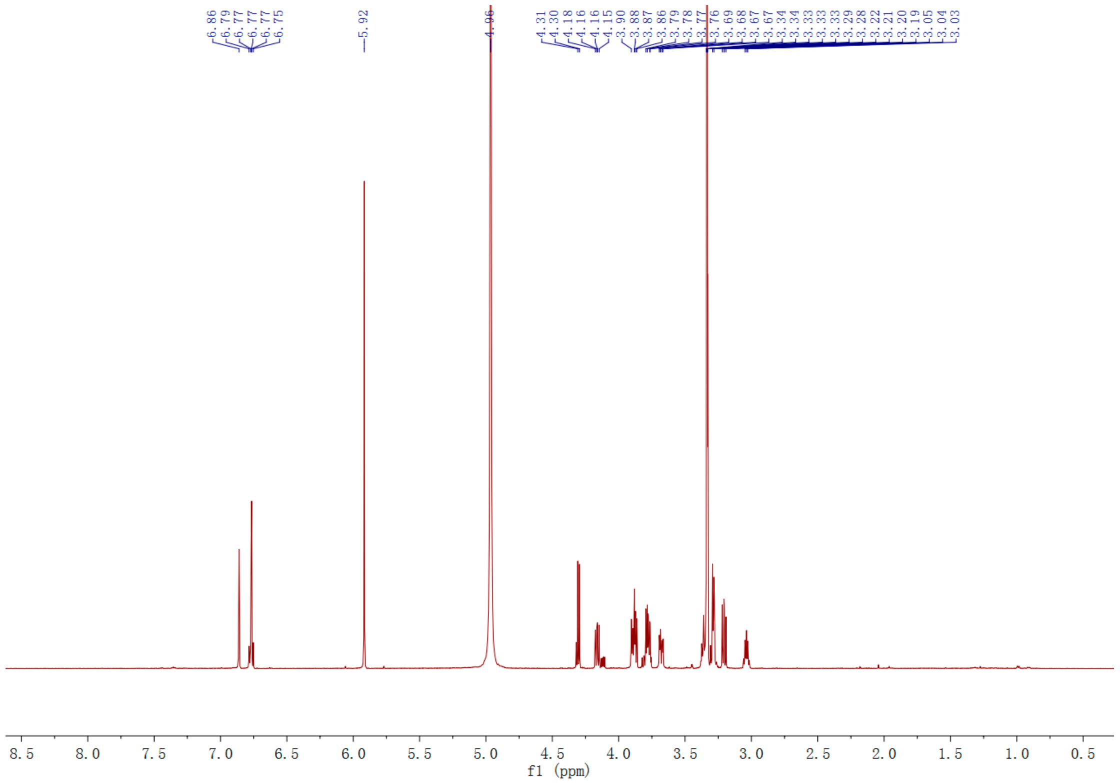

Figure S25. ^{1}H-NMR spectrum of 1-β-D-glucosyloxy-2-(3,4-methylenedioxyphenyl) -propane -l,3-diol (**9**) in CD_3OD.

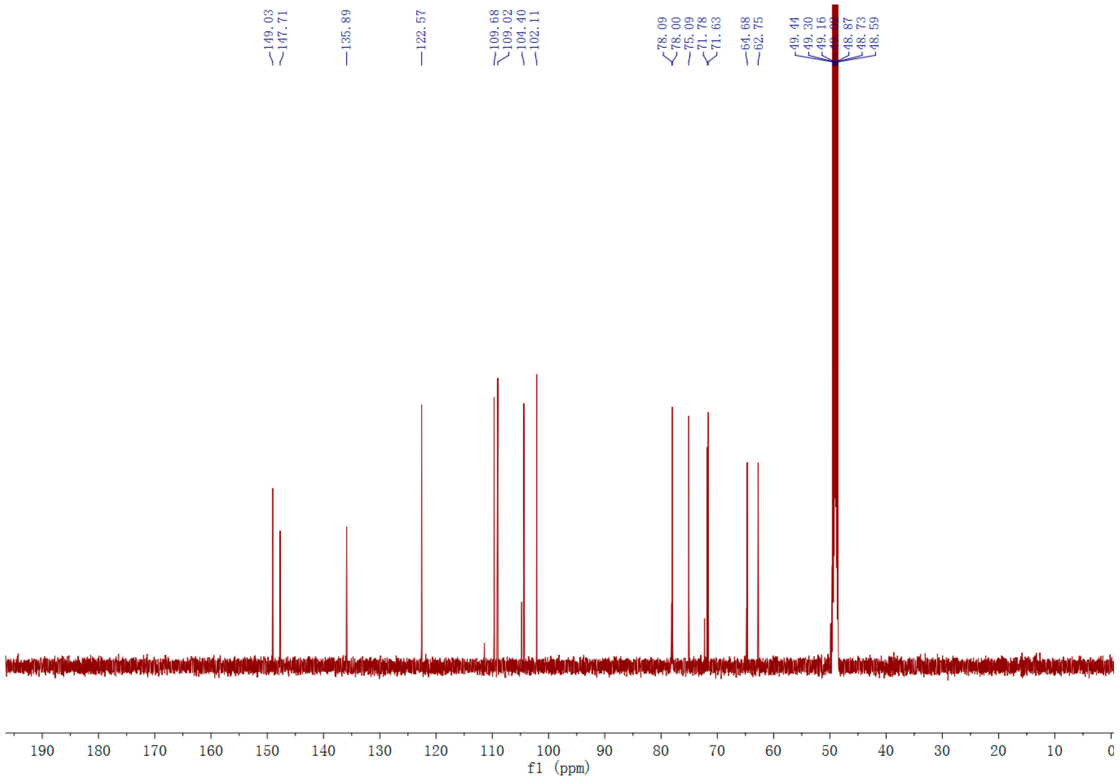

Figure S26. ^{13}C-NMR spectrum of 1-β-D-glucosyloxy-2-(3,4-methylenedioxyphenyl) -propane -l,3-diol (**9**) in CD_3OD.

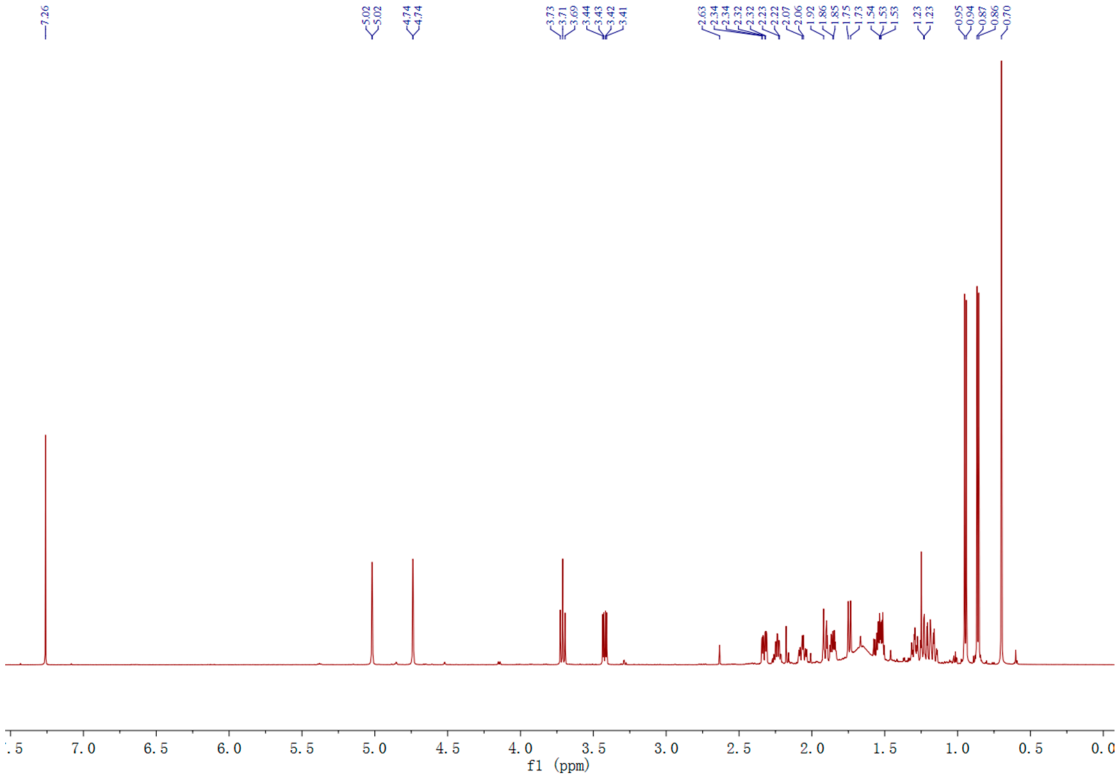

Figure S27. ^{1}H-NMR spectrum of lβ,6α-dihydroxy-4(14)-eudesmene (**10**) in CDCl3.

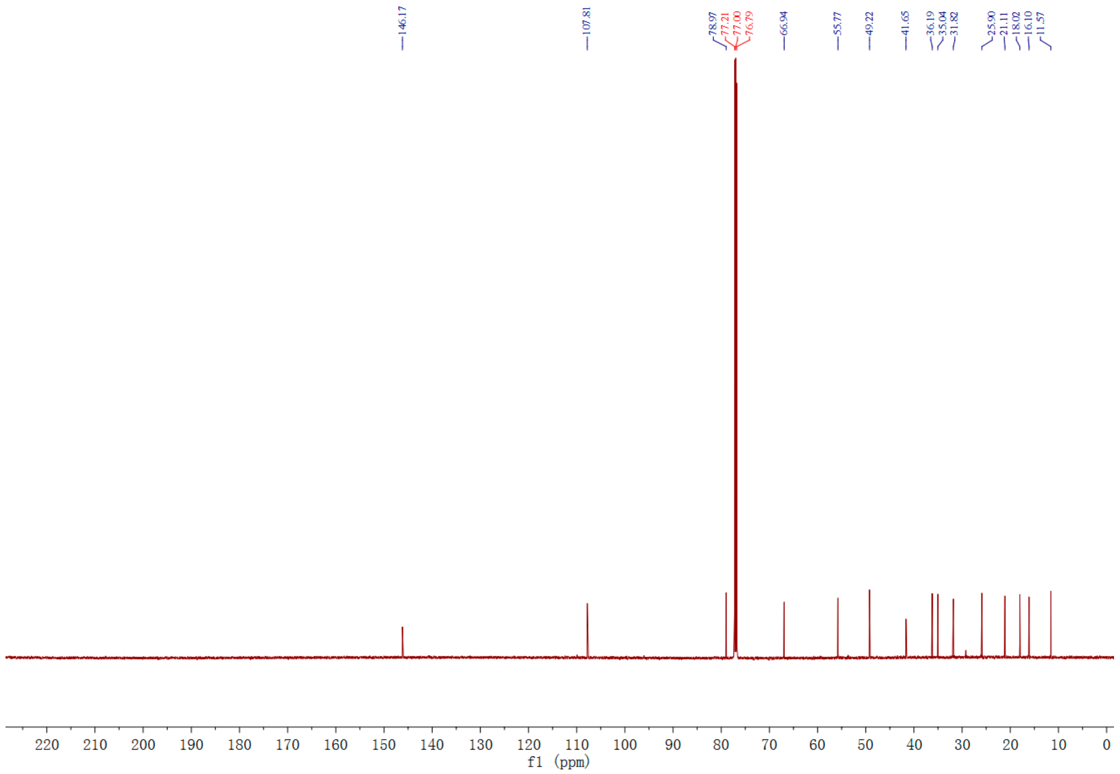

Figure S28. ^{13}C-NMR spectrum of lβ,6α-dihydroxy-4(14)-eudesmene (**10**) in $CDCl_3$.

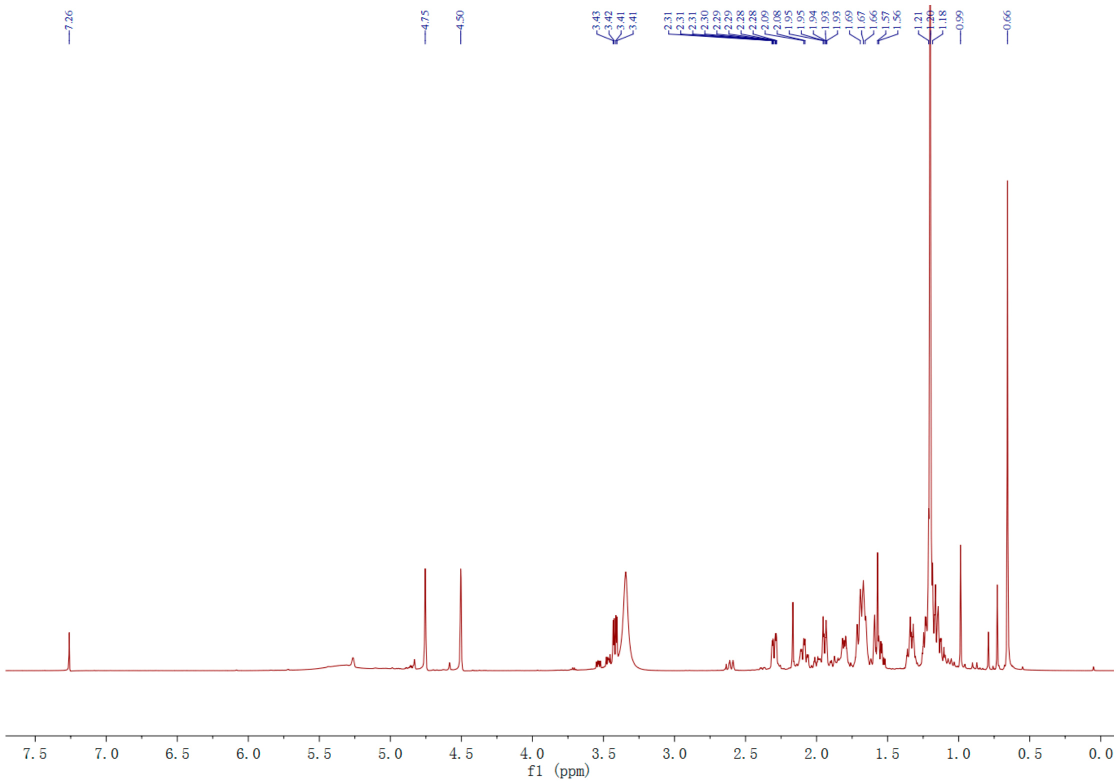

Figure S29. ^{1}H-NMR spectrum of selin-4(15)-en-1β,11-diol (**11**) in $CDCl_3$.

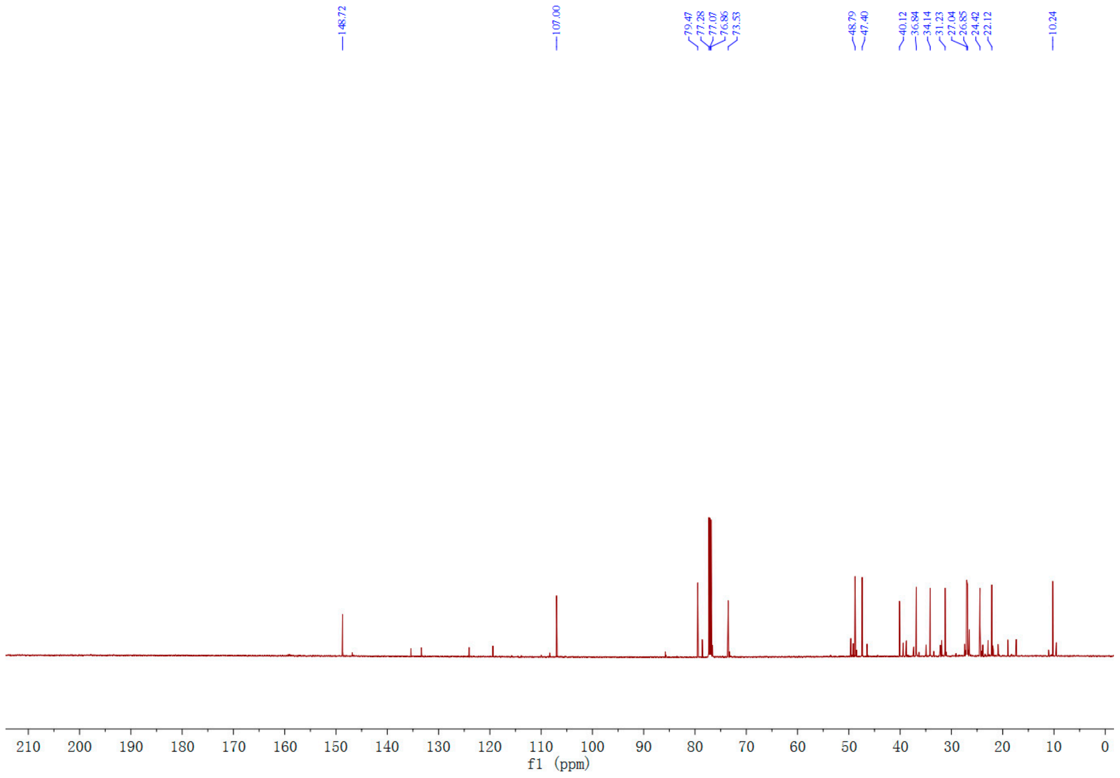

Figure S30. ^{13}C-NMR spectrum of selin-4(15)-en-1β,11-diol (**11**) in $CDCl_3$.

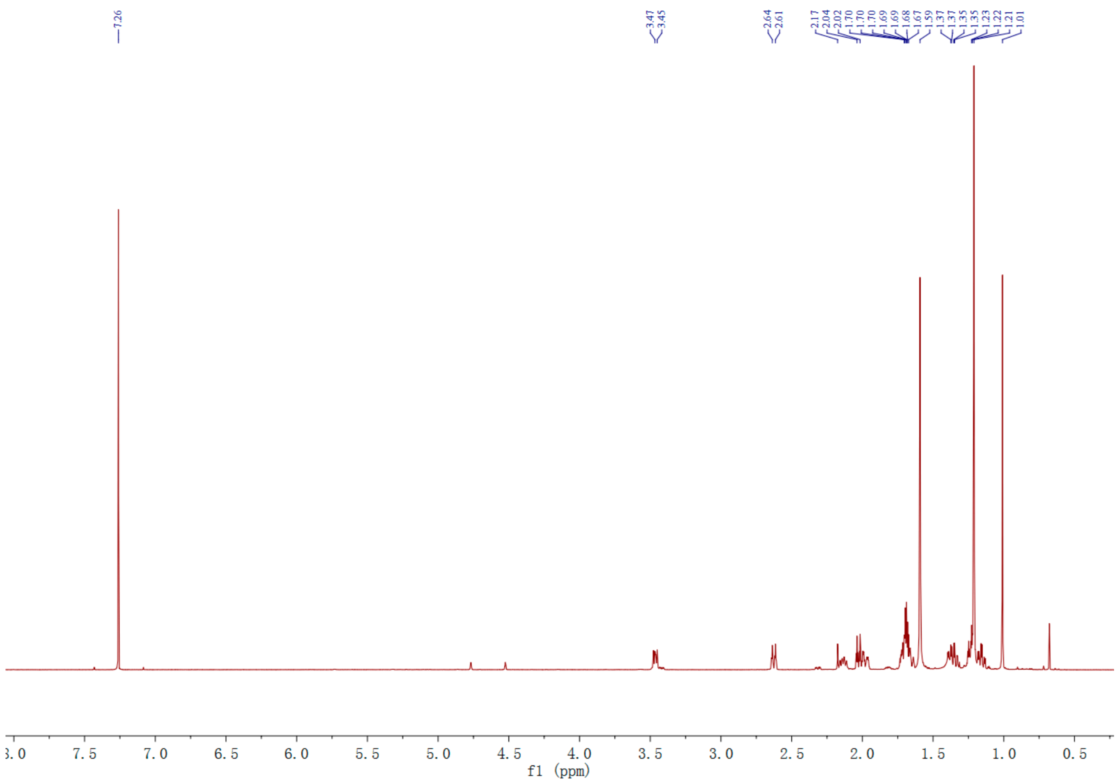

Figure S31. ^{1}H-NMR spectrum of 4-eudesmene-1β,11-diol (**12**) in $CDCl_3$.

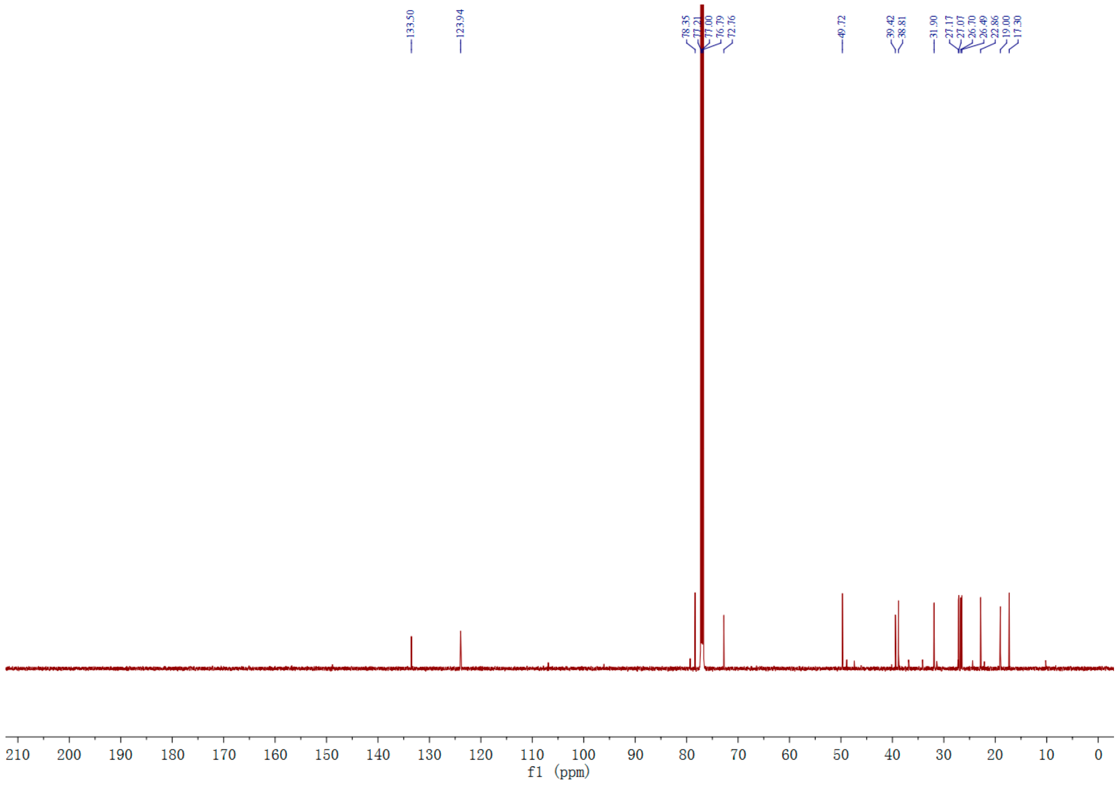

Figure S32. ^{13}C-NMR spectrum of 4-eudesmene-1β,11-diol (**12**) in $CDCl_3$.

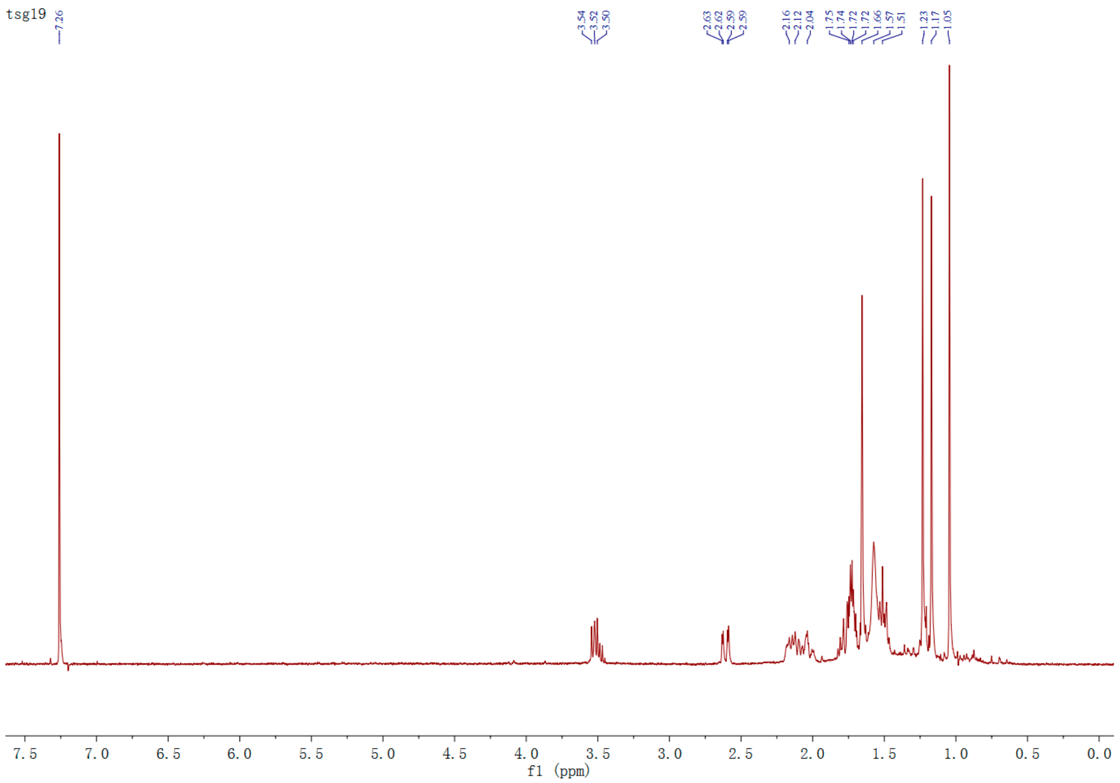

Figure S33. ^{1}H-NMR spectrum of 7-*epi*-4-eudesmene-1β,11-diol (**13**) in $CDCl_3$.

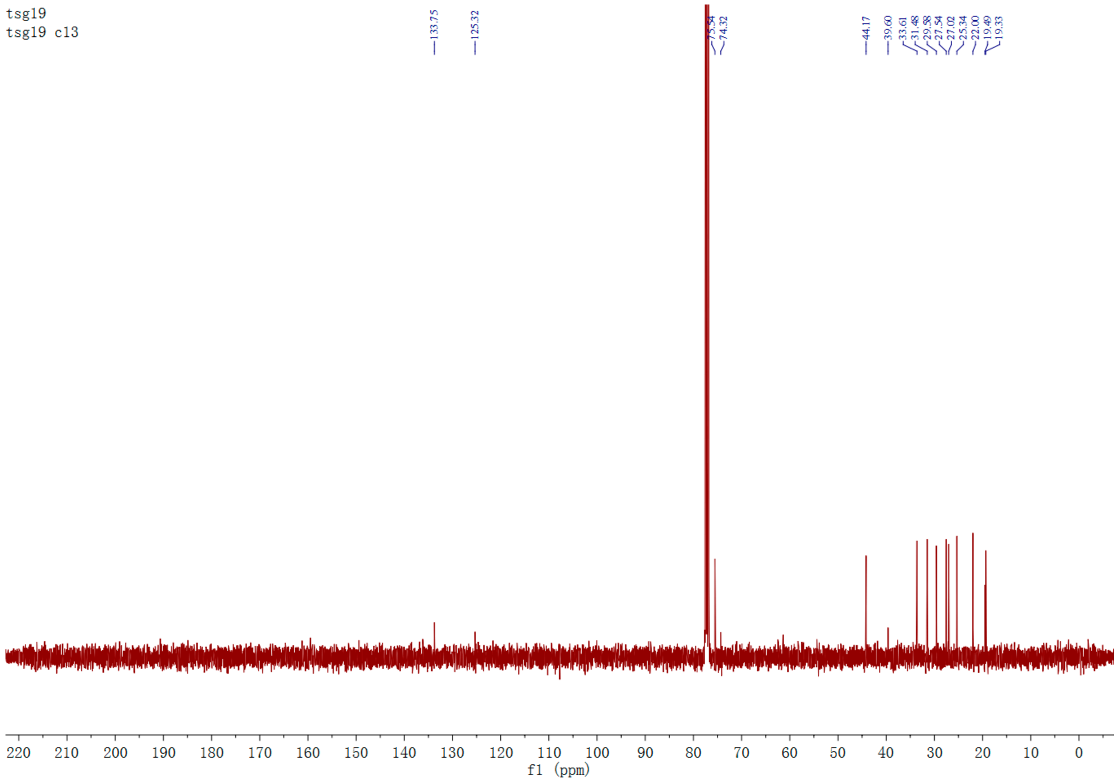

Figure S34. ^{13}C-NMR spectrum of 7-*epi*-4-eudesmene-1β,11-diol (**13**) in $CDCl_3$.

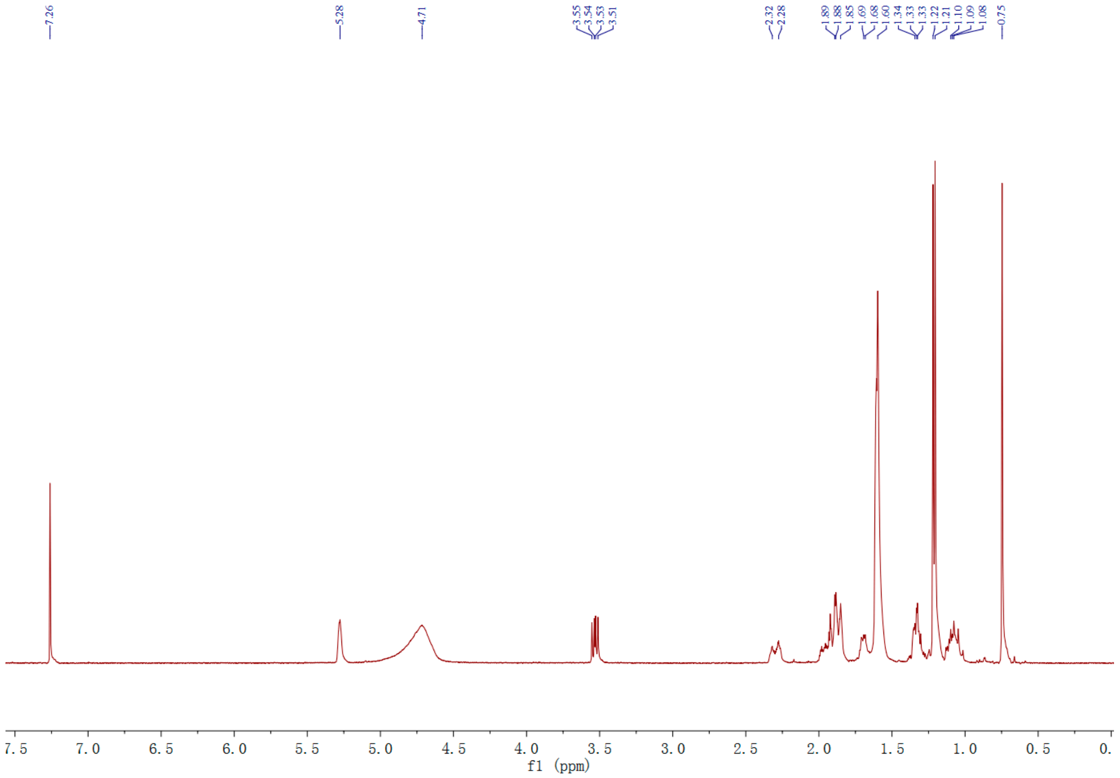

Figure S35. ^{1}H-NMR spectrum of 3-eudesmene-1β,11-diol (**14**) in $CDCl_3$.

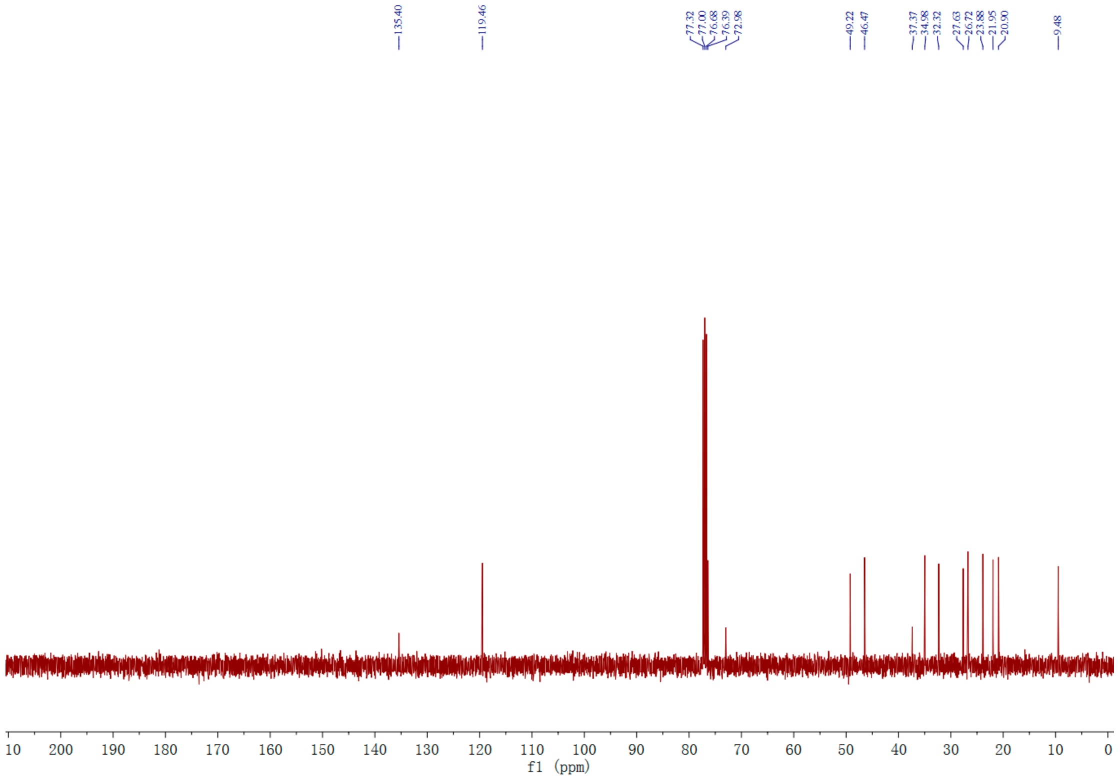

Figure S36. ^{13}C-NMR spectrum of 3-eudesmene-1β,11-diol (**14**) in $CDCl_3$.

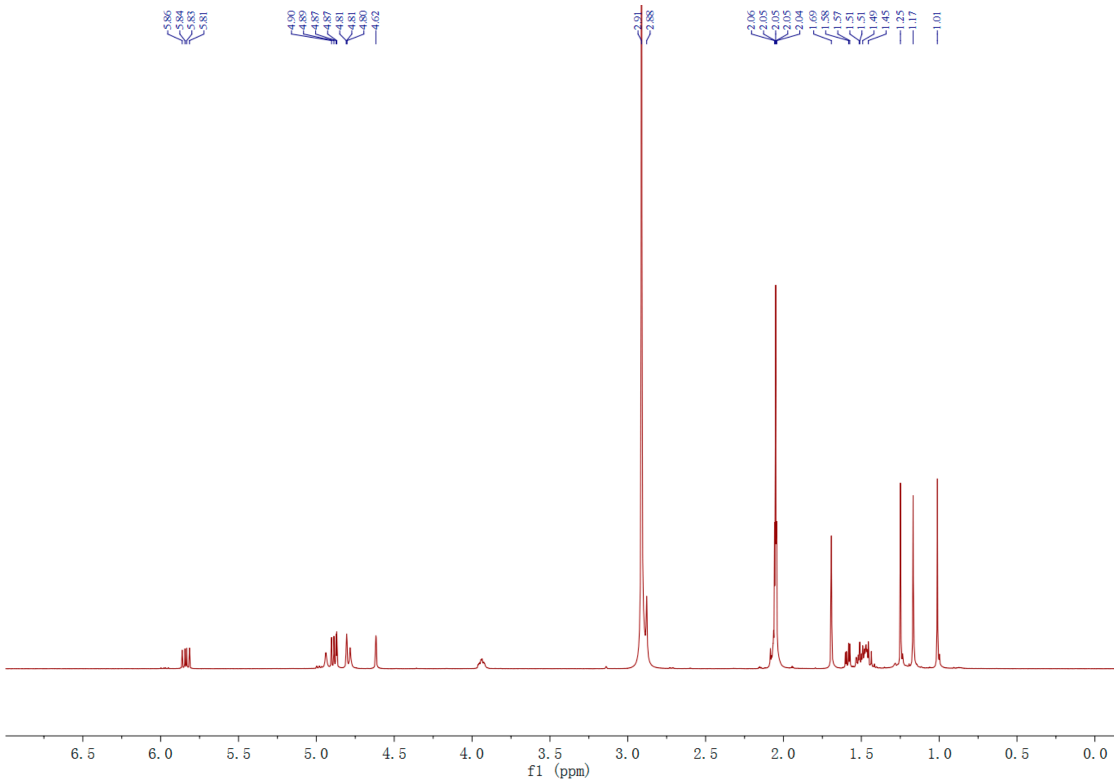

Figure S37. ^{1}H-NMR spectrum of 8α,11-elemodiol (**15**) in acetone-*d*6.

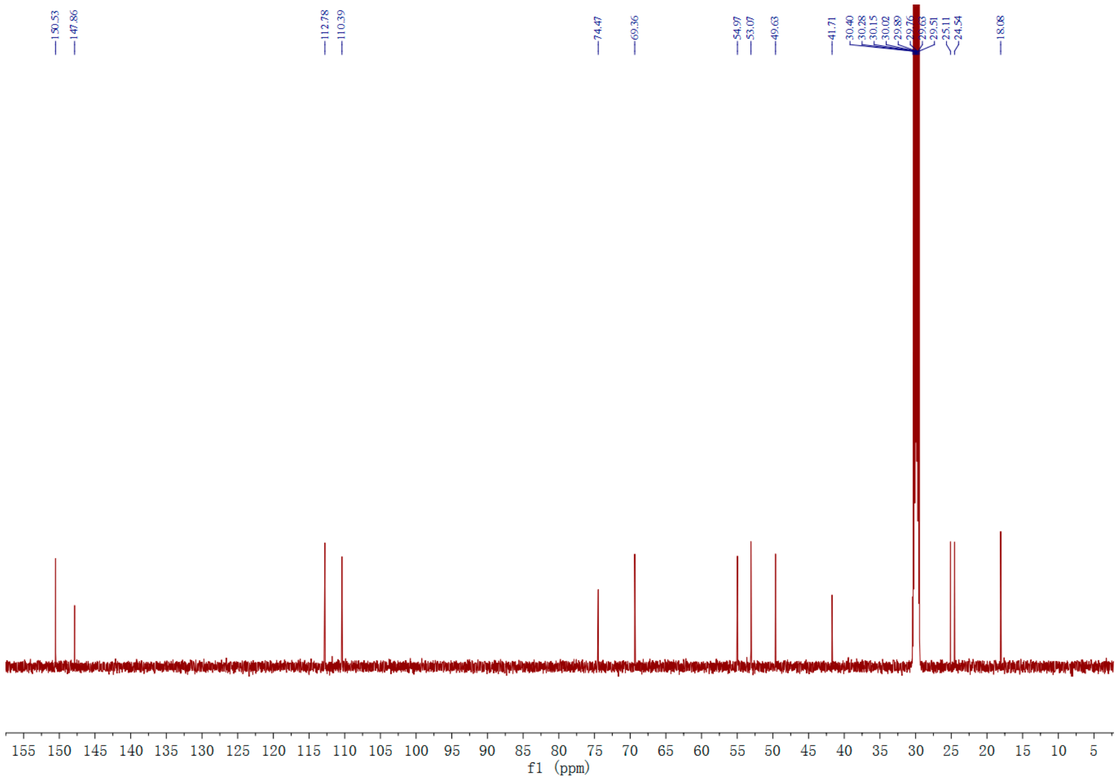

Figure S38. ^{13}C-NMR spectrum of 8α,11-elemodiol (**15**) in acetone-*d*6.

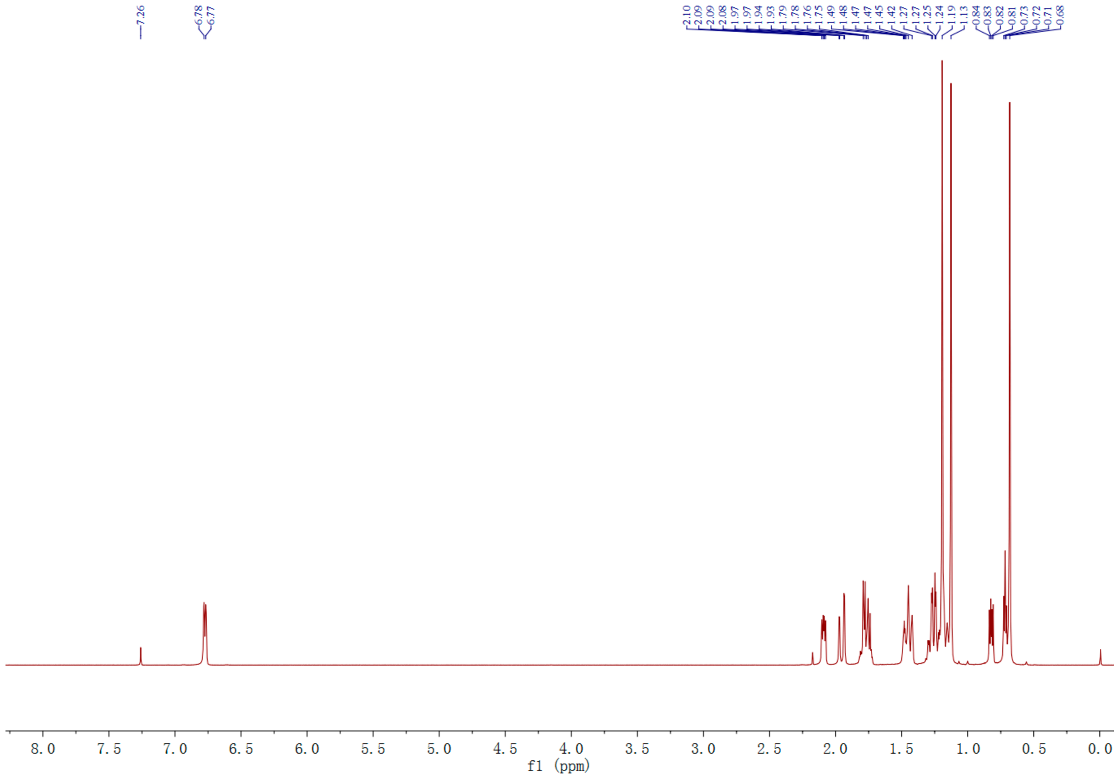

Figure S39. ^{1}H-NMR spectrum of hinokiic acid (**16**) in $CDCl_3$.

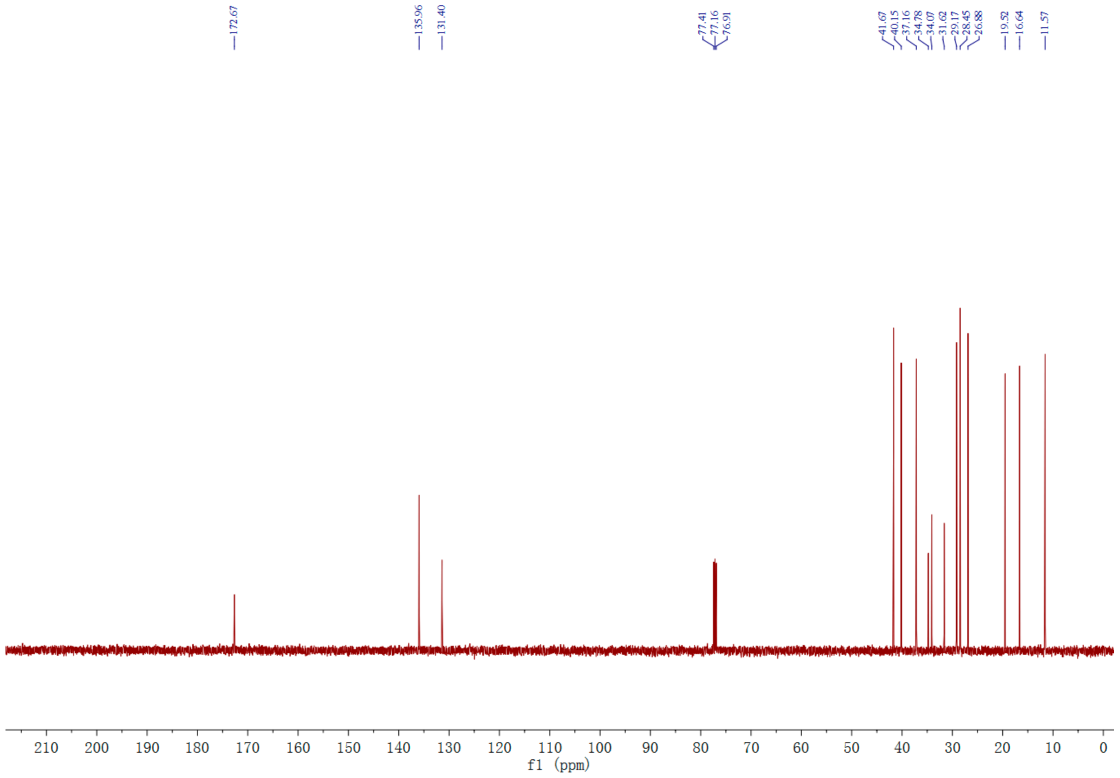

Figure S40. ^{13}C-NMR spectrum of hinokiic acid (**16**) in $CDCl_3$.

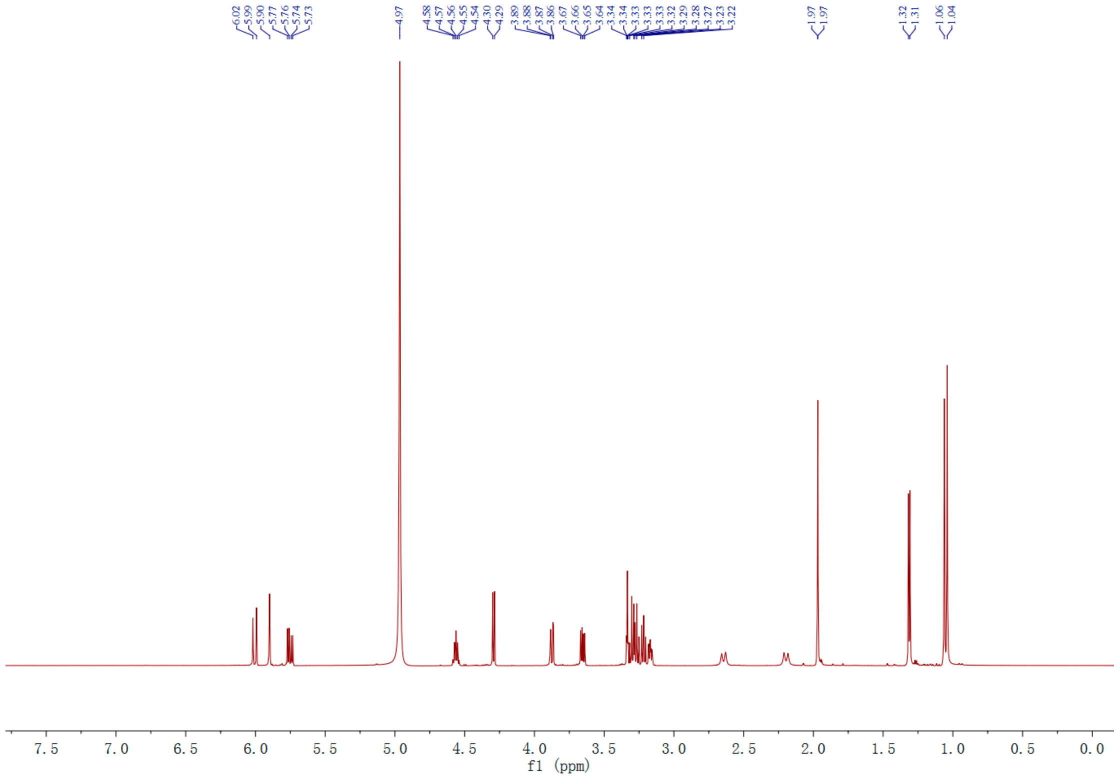

Figure S41. ^{1}H-NMR spectrum of corchoionoside C (**17**) in CD_3OD.

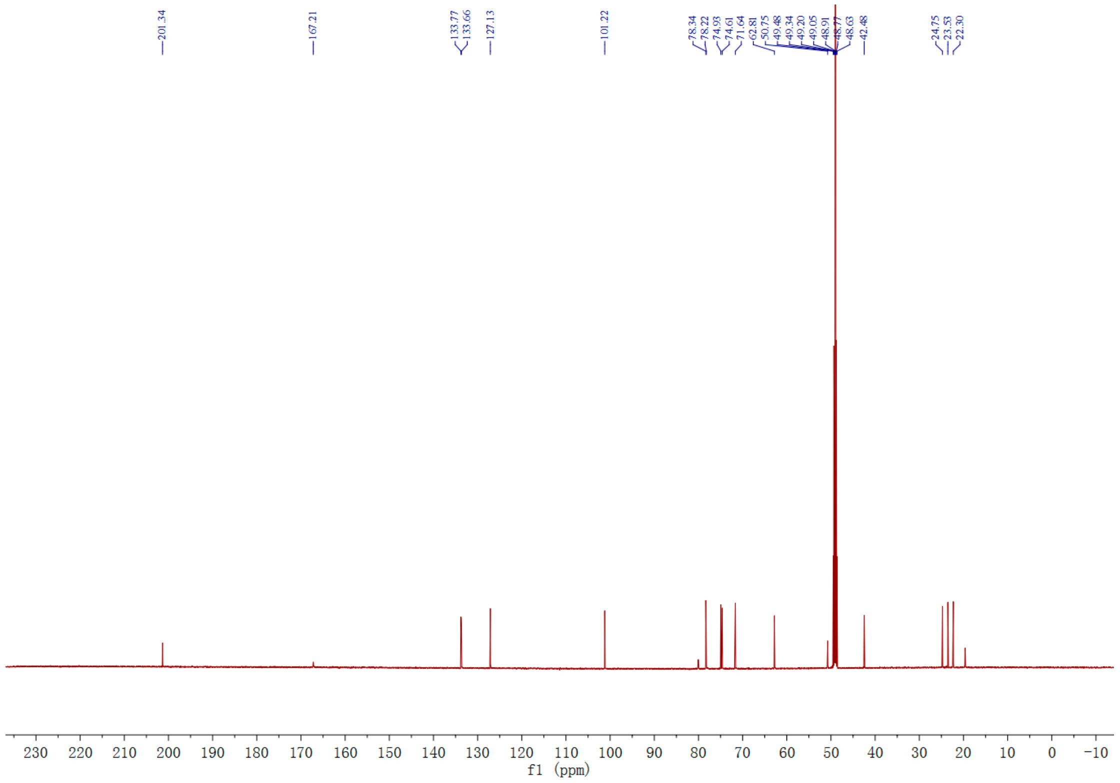

Figure S42. ^{13}C-NMR spectrum of corchoionoside C (**17**) in CD_3OD.

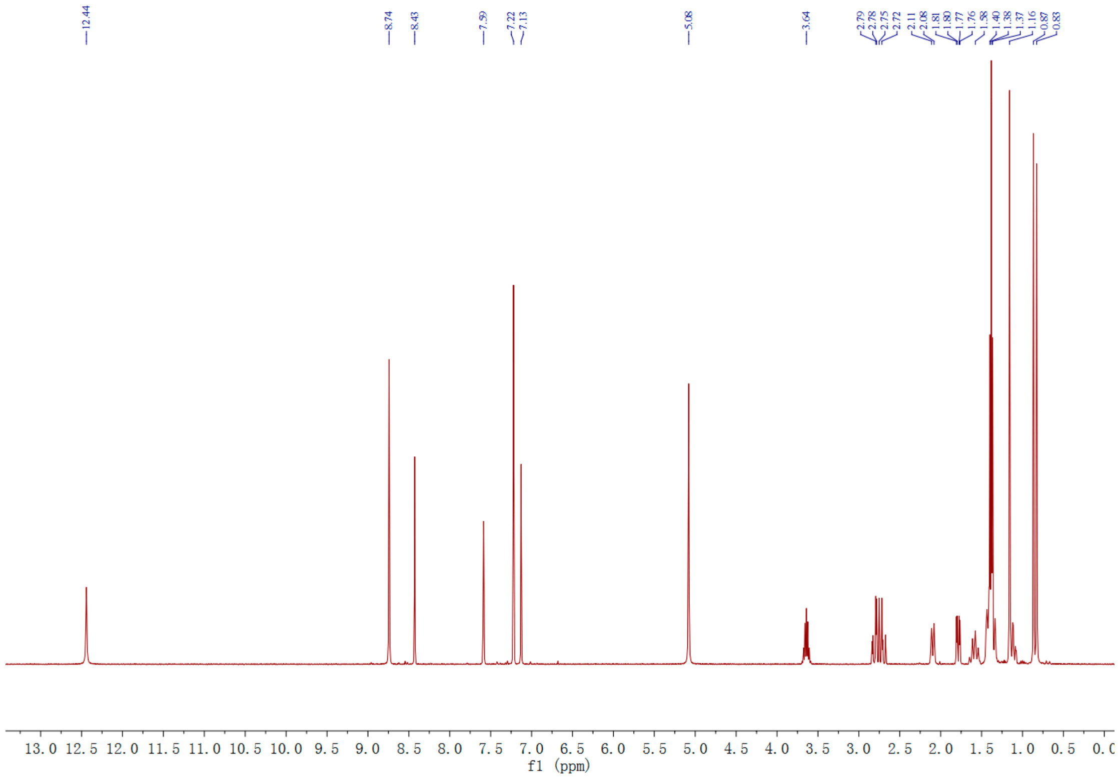

Figure S43. ^{1}H-NMR spectrum of hinokiol (**18**) in pyridine-d_6.

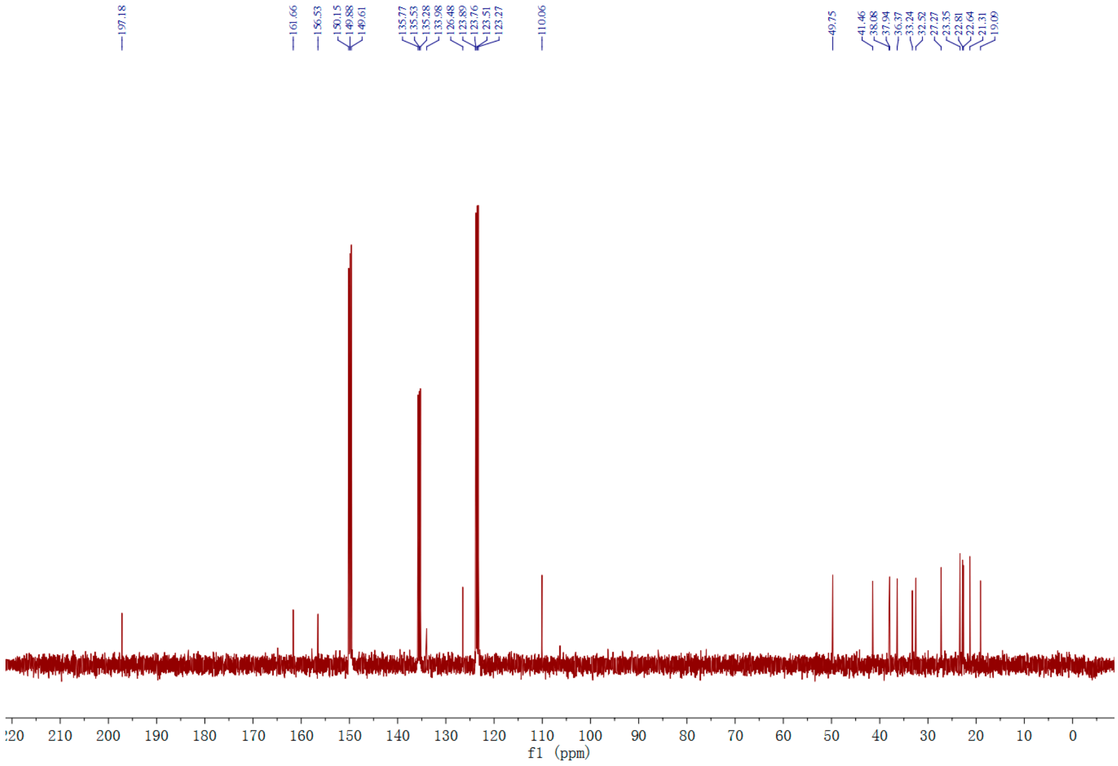

Figure S44. ^{13}C-NMR spectrum of hinokiol (**18**) in pyridine-d_6.

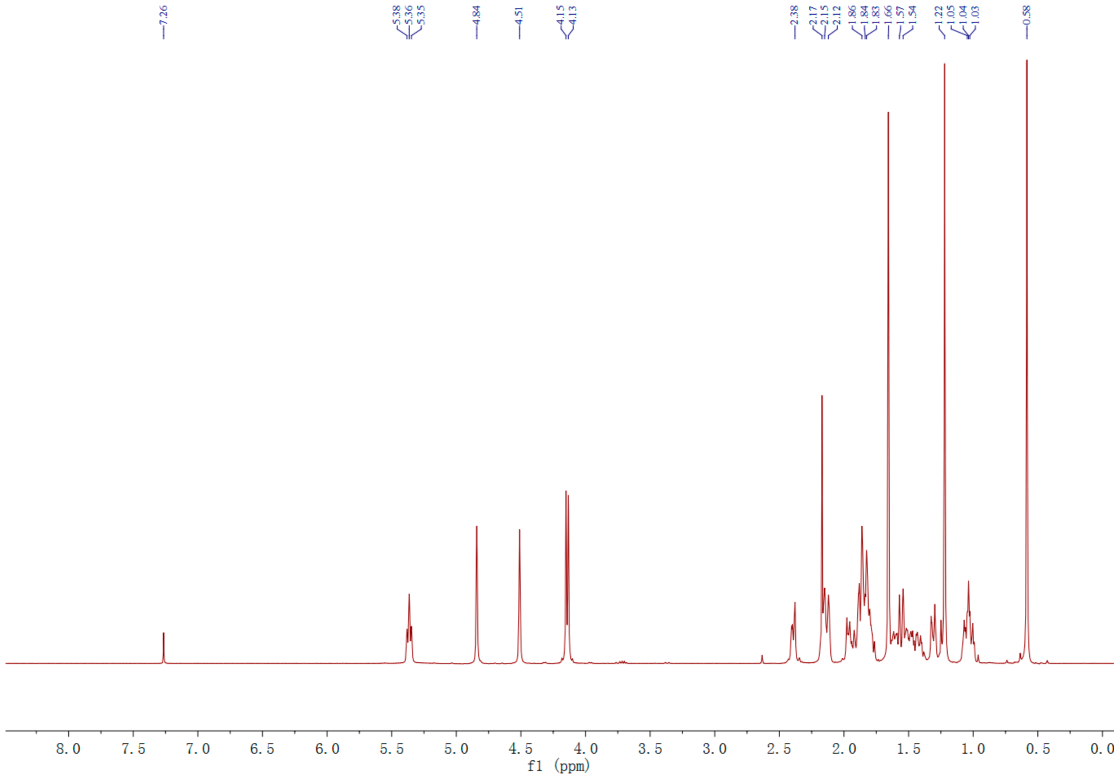

Figure S45. ^1H-NMR spectrum of isocupressic acid (**19**) in $CDCl_3$.

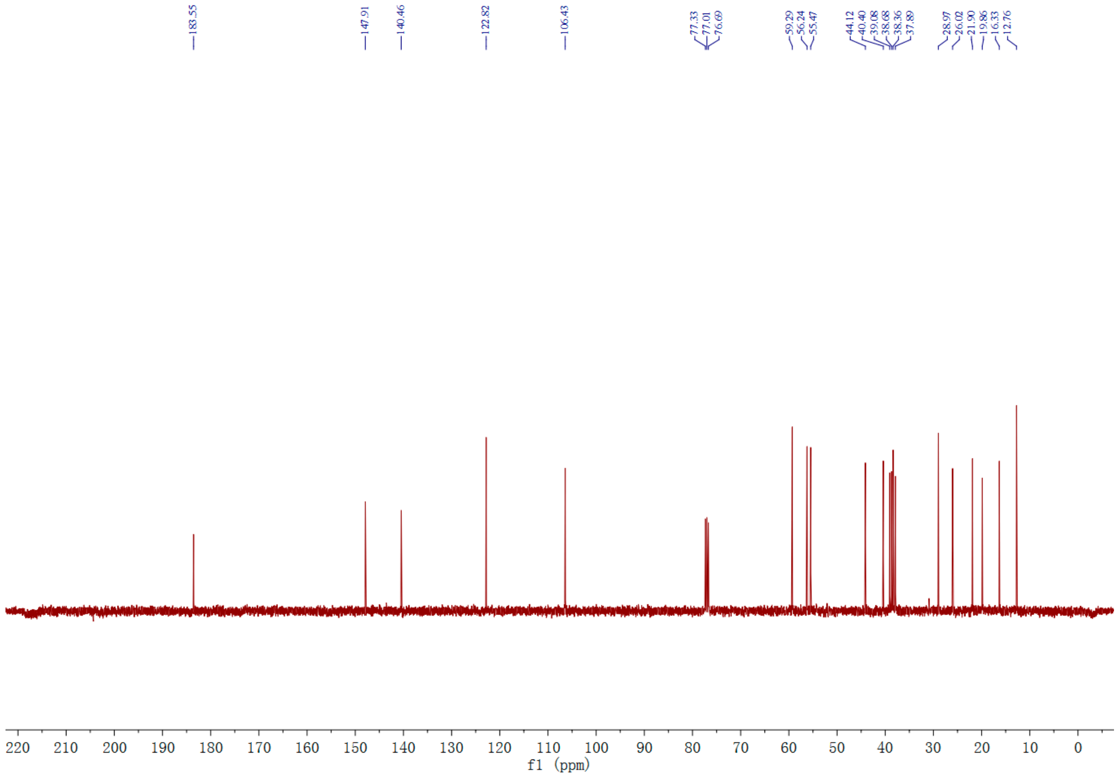

Figure S46. ^{13}C-NMR spectrum of isocupressic acid (**19**) in $CDCl_3$.

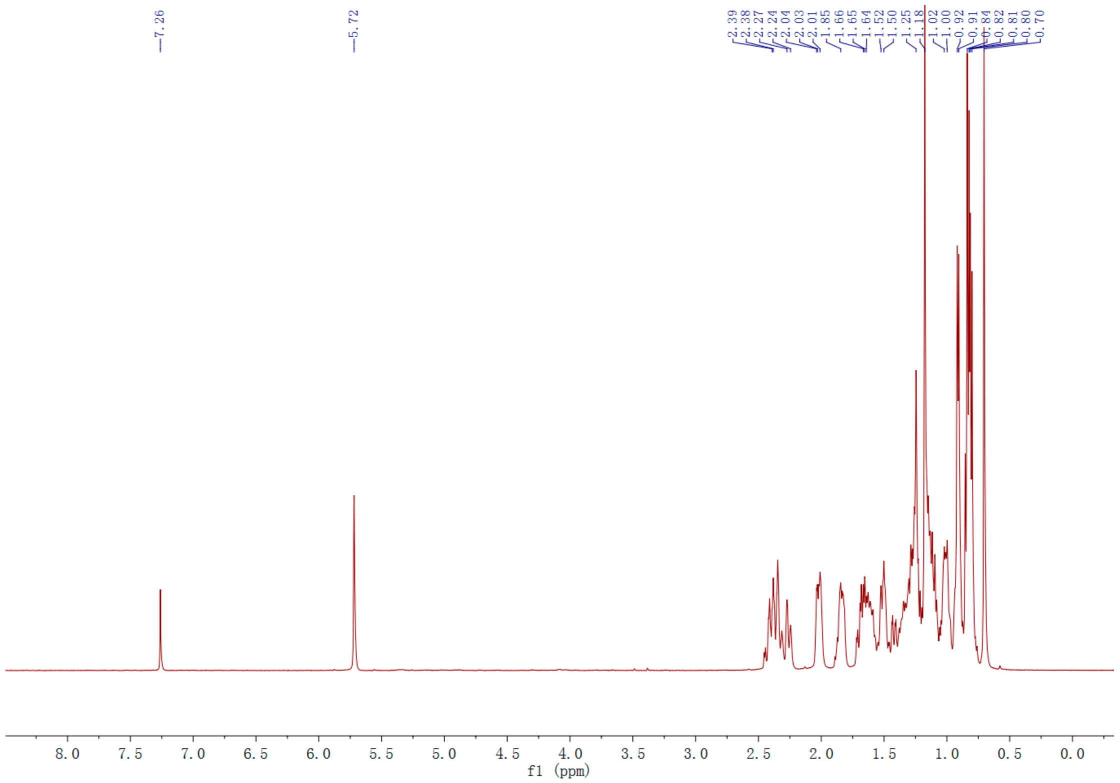

Figure S47. ^{1}H-NMR spectrum of sitostenone (**20**) in $CDCl_3$.

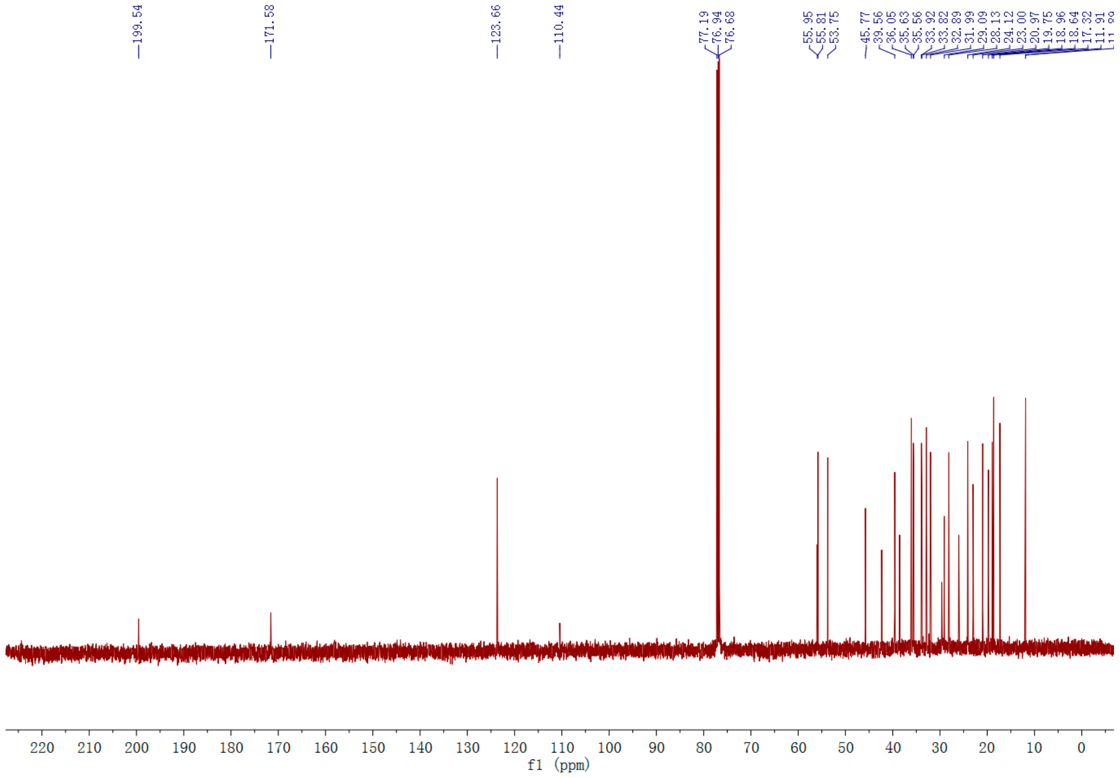

Figure S48. ^{13}C-NMR spectrum of sitostenone (**20**) in $CDCl_3$.
